# Bio::Homology::InterologWalk - A Perl module to build putative protein-protein interaction networks through interolog mapping

**DOI:** 10.1186/1471-2105-12-289

**Published:** 2011-07-18

**Authors:** Giuseppe Gallone, T Ian Simpson, J Douglas Armstrong, Andrew P Jarman

**Affiliations:** 1 Centre for Integrative Physiology, University of Edinburgh. Hugh Robson Building, George Square, Edinburgh EH8 9XD, UK; 2 Institute for Adaptive and Neural Computation, University of Edinburgh. 10 Crichton Street, Edinburgh, EH8 9AB, UK

## Abstract

**Background:**

Protein-protein interaction (PPI) data are widely used to generate network models that aim to describe the relationships between proteins in biological systems. The fidelity and completeness of such networks is primarily limited by the paucity of protein interaction information and by the restriction of most of these data to just a few widely studied experimental organisms. In order to extend the utility of existing PPIs, computational methods can be used that exploit functional conservation between orthologous proteins across taxa to predict putative PPIs or 'interologs'. To date most interolog prediction efforts have been restricted to specific biological domains with fixed underlying data sources and there are no software tools available that provide a generalised framework for 'on-the-fly' interolog prediction.

**Results:**

We introduce Bio::Homology::InterologWalk, a Perl module to retrieve, prioritise and visualise putative protein-protein interactions through an orthology-walk method. The module uses orthology and experimental interaction data to generate putative PPIs and optionally collates meta-data into an Interaction Prioritisation Index that can be used to help prioritise interologs for further analysis. We show the application of our interolog prediction method to the genomic interactome of the fruit fly, *Drosophila melanogaster*. We analyse the resulting interaction networks and show that the method proposes new interactome members and interactions that are candidates for future experimental investigation.

**Conclusions:**

Our interolog prediction tool employs the Ensembl Perl API and PSICQUIC enabled protein interaction data sources to generate up to date interologs 'on-the-fly'. This represents a significant advance on previous methods for interolog prediction as it allows the use of the latest orthology and protein interaction data for all of the genomes in Ensembl. The module outputs simple text files, making it easy to customise the results by post-processing, allowing the putative PPI datasets to be easily integrated into existing analysis workflows. The Bio::Homology::InterologWalk module, sample scripts and full documentation are freely available from the Comprehensive Perl Archive Network (CPAN) under the GNU Public license.

## Background

In recent years, large protein-protein interaction (PPI) datasets have allowed the description of relationships between proteins in complex biological systems [1]. These data are commonly derived from yeast two hybrid (Y2H), co-immunoprecipitation or tandem affinity purification (TAP) assays and have been obtained from a variety of unicellular and multicellular organisms [2-6]. Recent advances in high resolution mass spectrometry have further contributed to the rapid accumulation of PPI data [7-9]. Unfortunately, large scale experimental discovery of PPIs remains difficult, expensive and beyond the means of many experimentalists. Currently, PPI data is almost exclusively limited to a few popular model organisms and amongst these coverage of the captured interactions is often biased to a particular domain and incomplete. For many organisms, PPI data lags behind or is non-existent compared with genome sequence data.

In an attempt to address the relative paucity of data, a number of computational techniques have been proposed to predict and prioritise PPIs [10,11]. While the number of such methods is large, we focus here on methods that transfer functional information using cross-species orthology projection [12,13]. In essence, the rationale is that for interacting proteins *x *and *y *in organism  we expect (under certain conditions) that their orthologues *x*' and *y*' in organism  will also interact. Such conserved interaction pairs are called 'interologs' [14,15]. The potential use of interolog mapping has been explored in several organisms including *Homo sapiens *[16-22], *Helicobacter pylori *[23], *Saccharomyces cerevisiae *[24], *Plasmodium falciparum *[25] and *Magnaporthe grisea *[26]. Additionally a number of quantification methods have been developed to assess the confidence of predicted interologs [27-29].

Several web interfaces to interolog databases have been developed (e.g. HomoMINT [19] and Ulysses [20]), but these are essentially *ad hoc *efforts. They consider a small set of organisms for interolog prediction in restricted biological domains, thus hindering more widespread use. Underlying data sets are often frozen at the moment of publication or curated for a limited period of time (e.g. InteroPORC [28]), are dependent on other projects that are not updated (e.g. Integr8 [30]) or are based on algorithms that are not state-of-the-art. Considering that both orthology projection methods and interaction data are continually updated, such static databases are destined to obsolescence. To date the only project that provides the option to discover interologs with up to date data is OpenPPI predictor [31]. This represents a step forward, but relies on the user providing both the orthology relationships and known PPI data and only performs mappings between two species, without ranking or prioritising the putative PPI network obtained.

To address the lack of tools for performing multi-species interolog prediction 'on the fly' we created the Perl module Bio::Homology::InterologWalk. The tool relies on BioPerl [32], the Ensembl Perl Core and Compara APIs [33,34] and the EBI-Intact PSI Common Query InterfaCe (PSICQUIC) enabled web service [35,36] for its operation. Bio::Homology::InterologWalk is freely available under the GNU General Public Licence at the Comprehensive Perl Archive Network (CPAN) [37,38].

Bio::Homology::InterologWalk accepts as input a list of Ensembl gene accession numbers from any of the vertebrate or metazoan genomes in Ensembl and also for all species in the Ensembl pan-taxonomic Compara database. The tool searches the Ensembl Core and Compara databases and the PSICQUIC-enabled EBI-Intact PPI database to collect and analyse gene orthology and PPI data, together with ancillary information. It then provides the option of filtering the putative interactions to retain those with strong experimental or phylogenetic support. In addition the user can query the PPI database directly to collect all known interactions for the input gene list. This allows the ready comparison of putative PPIs from interolog projection to known PPIs. The software outputs plain text tab-separated files and can also output network representations of the PPI data and their attributes in a format compatible with the widely used biological network analysis tool Cytoscape [39].

We demonstrate the use of the software to investigate the potential of interolog projection on the genome of the fruit fly, *Drosophila melanogaster *[40]. The analysis (a) generates a novel putative PPI network that strengthens the connectivity of the known PPI network (b) proposes new interaction candidates. We further calculate an Interaction Prioritisation indeX (IPX) for each of the PPIs and use these to create a sub-network centred on a core of 10 DNA replication proteins.

## Implementation

### Overview

**Figure 1:**
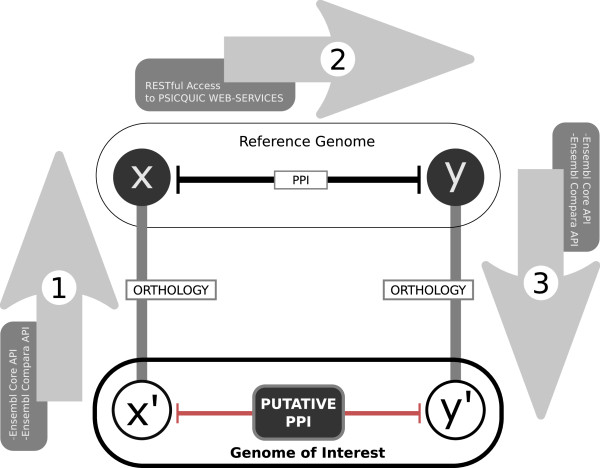
**The ****Bio::Homology::InterologWalk**** concept**. Schematics illustrating the principle behind interolog mapping. Proteins *x *and *y *are known to interact in a reference genome. If they have orthologues *x*' and *y*' in the genome of interest, under certain conditions the existence of a putative *x*' *- y*' interaction can be assumed. Bio::Homology::InterologWalk implements this in a three-steps algorithm. **1**. *get orthologues of genes of interest in reference genome(s)*. queries the initial gene list against one or more Ensembl databases and retrieves their orthologues. Options can be set to specify stringency of retrieved hits. Ancillary data fields are computed. **2**. *get interactions in reference genome(s)*. queries the orthology list built in (1.) against PSICQUIC-enabled PPI databases using REST. This step will enrich the dataset built in (1.) with the interactors of those orthologues, if any, plus ancillary data -- including parameters describing the nature and origin of the annotated interaction. **3**. *get orthologues from reference genome(s) back in genome of interest*. In this step the interactor list built in (2.) is queried against one or more Ensembl databases (again using the Ensembl Perl API) to find orthologues back in the original genome of interest. As in (1.), a number of supplementary information fields are computed.

A high-level schematic describing our implementation of the interolog walk concept is shown in Figure 1. The main purpose of Bio::Homology::InterologWalk is to obtain a list of putative PPIs given a set of user-selected gene identifiers in one genome of interest. In order to be compatible with the module, the initial dataset must be a list of Ensembl IDs belonging to species in Ensembl Vertebrates, EnsemblGenomes Metazoa or Ensembl Pan-taxonomic Compara databases.

**Figure 2:**
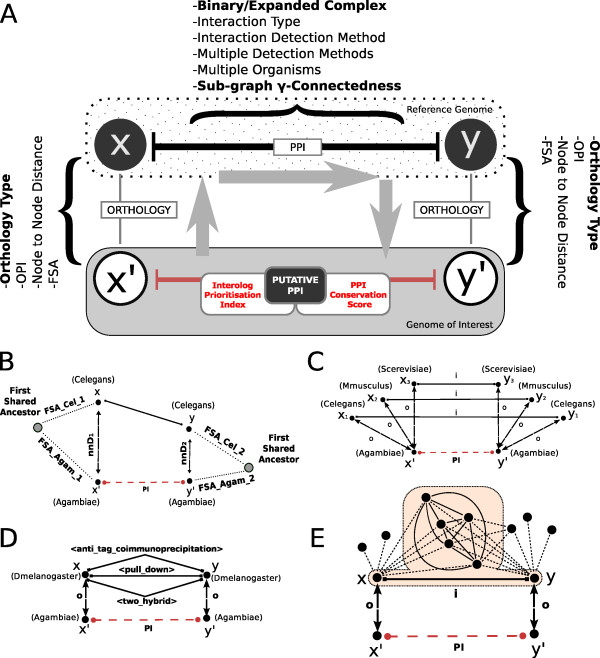
**Supplementary Data Fields & Prioritisation Features**. Schematics summarizing the features used to prioritise the resulting putative PPIs. For each PPI, a number of metadata fields are collected during the three main steps of the algorithm. Two metrics can optionally be computed: an *Interolog Prioritisation Index *(IPX) and a *PPI Conservation Score *(PCS) (Additional File 2 - Definitions). **A**. Overall view showing the contributing data fields. **B-E**. Sample prioritisation features. **B**: Phylogenetic distances (according to TreeBeST). For each of the two orthologous pairs, a node-to-node distance (nnD*_i_*) and two distances from the First Shared Ancestor  are computed. **C**: experimental interaction observed in multiple taxa - a component of the IPX is proportional to the number of reference genomes contributing to a putative PPI evidence. **D**: experimental interaction reconfirmed through multiple detection methods - a component of the IPX is proportional to the number of detection methods used to obtain experimental PPI evidence in the reference genome. **E**: PPI Conservation Score. The conservation score depends on (1) the density of the most-connected *γ *-clique that includes *x*, *y *and their mutual interactors and (2) the number of edges of the *γ *clique.

To carry out an interolog walk, Bio::Homology::InterologWalk will first query the gene identifiers chosen by the user against the Ensembl databases using the Ensembl Compara API [41], retrieving a list of orthologous gene IDs. Next, the algorithm will use the Representational State Transfer (RESTful) interface [42] to interrogate a PSICQUIC-compliant PPI database with the list of orthologues returned by Ensembl, to retrieve the list of known PPIs involving them. While there are already several interaction databases implementing the PSICQUIC interface [43-50], Bio::Homology::InterologWalk currently relies on EBI IntAct [36] as its source of experimental interactions. Having obtained a list of interactors for the orthologues of the initial gene set, in the last step of the main data mining procedure Bio::Homology::InterologWalk will project the interactions retrieved (again, using the Ensembl Compara API) back to the original species of interest. The final output is a list of putative interactors for the initial gene set and several fields of supporting data for the forward orthology map, the PPI data collection, and the backward orthology map. These metadata fields can be analysed by a sub-module of the tool, to calculate a prioritisation index for the predicted PPIs (Figure 2A).

The procedure is organised as a pipeline of related data-processing activities. The output of the basic pipeline can be further processed with the help of other methods in the module: it is possible to scan the results and compute counts, check for duplicate entries, isolate new gene IDs (not part of the original dataset) and generate Cytoscape-compatible network representations of the data. The package documentation gives a detailed account of post-processing options.

An additional stand-alone functionality of the module is the *direct *PPI pipeline: it is possible to use Bio::Homology::InterologWalk to mine all the experimental PPIs involving the initial gene list in the genome of interest (without mapping to reference genomes using orthology). This dataset is a 'snapshot' of the current experimental PPI network for the input dataset. As such it is useful both by itself, because it tells what is currently know about the PPIs involving the initial genes, and as a term of comparison for the putative PPI - because it can be used to evaluate the amount of overlap between the known and putative networks, as well as the novelty of the putative data. Additional file 1: 'Simplified schematics of the Interolog Walk Pipeline' illustrates both the putative and the experimental pipeline in detail.

One of the defining features of Bio::Homology::InterologWalk is that the retrieval of both orthology data and protein interaction data happens on-the-fly. The user inputs a list of gene IDs plus a number of set-up parameters, and the data will be downloaded through web-service interfaces each time the program is run.

### Data Sources

#### Orthology Predictions from Ensembl Compara

Bio::Homology::InterologWalk uses the Ensembl Perl API http://www.ensembl.org/info/data/api.html to access the comparative biology data provided by the Ensembl Project through Ensembl Compara. The orthology prediction method used by Ensembl Compara is described by Vilella *et al*. [41] and identifies several classes of homology association between genes (Additional file 2: 'Definitions').

#### PPI Data from EBI-IntAct

Bio::Homology::InterologWalk currently uses EBI IntAct [36] as its source of experimental interactions. As of June 2011, v. 1.1.7 of the IntAct database contains more than 267,000 curated binary protein interaction evidences [36]. Bio::Homology::InterologWalk queries Intact using the RESTful-based PSICQUIC [Aranda *et al*., in preparation] implementation and retrieves data in PSI-MI MITAB25 tab-delimited format [35] (Additional file 2: 'Definitions').

### Options for Prioritisation of Putative Interactions

#### Filtering

Depending on the size of the input dataset and on the amount of information available through homology mapping, Bio::Homology::InterologWalk can produce large numbers of putative interactions. In such cases it might be beneficial to filter and prioritise these to generate a smaller set of putative interactions for further study. The Bio::Homology::InterologWalk module is composed of a number of functions that can be executed in sequence to create pipelines for retrieving interologs. Each of these functions offers options to filter the types of orthologues and interactions that are retrieved.

1. *Spoke interactions*: the user can choose whether to return any 'spoke' interactions when using interaction retrieval functions. Spoke interactions are binary interactions that are inferred from a complex of proteins that have been isolated together and as such the evidence for the interaction is indirect. Several of the most widely used protein-protein interaction data repositories including the two largest IntAct and BioGrid explicitly draw the user's attention to the presence of spoke (or co-existence) interactions and provide the option of excluding them at an early stage.

2. *One-to-one orthology*: for each of the orthology mapping functions the user can choose whether to restrict the mapping to explicit 1:1 relationships. This is likely to significantly reduce the number of orthologues retrieved as the evolutionary distance between mapped species increases. Restricting mappings to direct orthologues increases the likelihood that the mapped proteins retain some common functionality. Conversely considering *1-to-many *or *many-to-many *relationships that have arisen through duplication events risks connecting proteins and interactions whose functions have diverged [51,52].

3. *Experimental interactions*: the user can specify whether to restrict the interactions retrieved to those that have been identified by experimental methods rather than by inference or prediction (Additional File 2: 'Definitions').

4. *Physical interactions*: the user can choose to retrieve only those interactions that test for direct physical association between proteins (Additional File 2: 'Definitions').

#### Prioritisation

We have created an *Interaction Prioritisation indeX *(IPX) and a *PPI Conservation Score *(PCS) that can optionally be used to aid in the prioritisation of putative interactions.

The IPX summarises the contribution of several pieces of heterogeneous information that are collected during orthology projection and interaction retrieval. It is not intended to be a quantitative measure of interaction reliability, but rather an integration of biological information such as orthology type, phylogenetic distance (FSA), percentage identity (OPI), interaction type and detection method (including multi-method). This is similar to the work of Huang *et al*. [22] and Yu *et al*. [27]. Yu *et al*. used sequence similarity between the orthologous proteins to build a join similarity score, while Huang *et al*. proposed a scoring framework based on GO functional annotation, domain information, tissue specificity and sub-cellular localisation to rank interolog-based human putative PPIs obtained from six eukaryotes. Some of the indicators evaluated to create the IPX are:

•**Orthology Type**. The kind of orthology relationship existing between an ID in the genome of interest and its orthologue in the reference genome. This feature indicates if there is a one-to-one mapping of orthologues, or if in-paralogy events in one or both sides mean we are considering a one-to-many, many-to-one or many-to-many orthologous mapping. As explained in the filtering section, we particularly value putative PPIs where *both *orthology relationships are of the one-to-one kind. It has been shown [51] that gene duplication is correlated with sub-functionalisation and neo-functionalisation. When the two orthologous pairs in the interolog walk are of the one-to-one kind we set a boolean variable, Θ, to a non-negative value in the score. We set Θ = 0 otherwise.

•**Expanded Complex**. Indicates whether the binary interaction has been extracted from a complex using the spoke expansion model. A boolean non-negative term, ∑, is added to the score to reward each true binary interaction. ∑ = 0 for spoke-expanded binary interactions.

•**OPI**. Overall Percentage Identity. A numerical index representing the percentage identity of the *conserved *columns between the two orthology members' sequences. Given *N *total samples, we define a *Joint OPI *as the geometric mean of the two OPIs (forward and backward orthology projection)

•**Node to Node Distance**. A numerical indicator of the node-to-node distance in the consensus phylogenetic/species tree built by Ensembl Compara using Genetrees [41] (Figure 2B). We consider

where nnD_1 _is the node-to-node distance between the two orthologues in the forward projection, nnD_2_ is the node-to-node distance between the two orthologues in the backward orthology projection and we set

•**Interaction Type & Interaction Detection Method**. Features based on PSI-MI controlled vocabulary terms indicating, respectively, the type of interaction and the detection method used, within the HUPO PSI-MI hierarchy (Additional File 2: 'Definitions', Table S1).

•**PPI obtained with Multiple Methods & annotated in Multiple Organisms**. Experimental PPIs reconfirmed through the usage of further detection methods and/or observed in multiple reference genomes are acknowledged by this feature (Figures 2C and 2D).

Overall, the putative PPI **Interolog Prioritisation indeX **is(1)

In this expression,(2)

 agglomerates the terms relative to the PPI in the reference organism: *i *is a feature scoring the interaction type and *d *is a feature scoring the interaction detection method. *m*_dm _acknowledges those experimental PPIs present in the database more than once, with different detection methods (Figure 2D). *m*_taxa _is set to the number of reference genomes that possess an experimental interaction projecting back to the same putative PPI (Figure 2C). The four features are normalised to make sure their values are comparable. Normalisation constants are explained in Additional File 2: 'Definitions'. The terms relative to the two orthology projections are combined in **S**_ORT_:(3)

We set *ω_i_*,= *ω_o _*= 1. Optimisation of these two weights based on training data will allow to reward either the interaction component or the orthology component of the score to optimise performance on a case-by-case basis. Lastly, ∑ and Θ are boolean terms and we set ∑ = 0 whenever the putative PPI has been inferred from a binary PPI derived from a spoke-expanded complex (∑ = *n*, where *n >*0 is an integer, otherwise), while Θ = *n *whenever the putative PPI has been inferred based exclusively on one-to-one orthology paths (Θ = 0 otherwise).

∑ and Θ are boolean flags not normalised in the IPX expression. This is done to obtain a gross selection of putative PPI samples based on co-orthology/no co-orthology and spoke/no spoke information, prior to looking at other secondary metadata features. The value *n *was chosen to be the smallest integer bigger than the maximum spread of the distribution of the normalised IPX features. The IPX is composed of 6 features, **f **= [*i*, *d*, *m_dm_*, *m*_taxa_, **J**_OPI_, **J**_nnD_], where 0 ≤ *f*_i_ ≤ 1, ∀*_i _*∈ 1, ..., 6 and so *n *= 7.

Allowing Θ and ∑ to be one order of magnitude bigger than other IPX features means the IPX distribution will take a roughly three-modal shape, depending on the combinatorial values of ∑ and Θ, as follows:

1. ∑ = 0, Θ = 0 (*Low Tier*) - the experimental interaction is spoke-expanded and at least one of the two orthology projections is not one-to-one.

2. (∑ = *n*, Θ = 0) Θ (∑ = 0*; *Θ = *n*) (*Mid Tier*) - either the experimental interaction is spoke-expanded or at least one of the two orthology projections is not one-to-one.

3. ∑ = *n*, Θ = *n *(*High Tier*) - the experimental interaction is not expanded from a spoke-complex and the orthology projections are both one-to-one.

Visual inspection of the modes in the IPX distribution can be used as strategy to filter out different sets of putative PPIs, depending on the dataset considered and on the distribution of samples within the modes of the histogram. The choice of *n *provides good visual separation of the modes in the IPX distribution to facilitate inspection. We refer to the module code for further details.

The PPI Conservation Score (PCS) focuses on the potential for evolutionary conservation in the projected PPI by examining the density of the sub-network from which each experimental PPI is extracted. It has been shown that the connectivity of well-conserved proteins in PPI networks is negatively correlated with their rate of evolution [53,54] and, as a consequence, more connected proteins evolve at lower rate because they are subject to higher pressure to co-evolve with interacting proteins. The PPI conservation score quantifies the degree of connectivity of the sub-network to which each experimental (known) PPI used for the interolog walk participates (Figure 2E). A binary interaction part of a very well-connected sub-network in the reference genome is more likely to have retained its functional characterisation after the projection to the organism of interest. In our implementation, we follow the method suggested by Huang and colleagues [22], and define the PPI Conservation Score as(4)

where *γ *= 2 *· E/*[*N · *(*N - *1)] and *N *and *E *are, respectively, the number of nodes and edges in the sub-network. Since the *γ*-connectedness measure is biased towards maximally connected small sub-networks, is relaxed by weighting it with the number of edges *E*.

Schematics illustrating the IPX and PCS are shown in Figure 2A-D and 2E respectively, and a detailed description of both can be found in Additional File 2: 'Definitions'.

There are many ways that an interolog could be prioritised. We aim for Bio::Homology::InterologWalk to be compatible with a diverse range of data and useful for many different kinds of users. Any prioritisation metric will be context-dependent and for this reason we offer a number of options to configure the process to suit the users requirements and the coverage and quality of the data available to them. As such the generalised and customisable prioritisation scheme we provide here should provide the necessary exibility to allow application across a broad range of biological domains.

## Results and Discussion

### Validation

#### Retrieving known interactions through orthology walking

**Figure 3:**
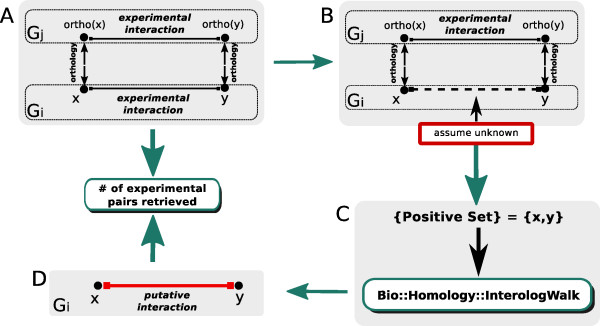
**Validation Procedure**. Schematics showing the rationale for the creation of the known positive sets  for validation. (**A**) Complete PPI datasets for two genomes *G_i _*and *G_j _*are retrieved. Only PPIs conserved across the two species through orthology are retained. PPI pairs in *G_i _*satisfying this property constitute the known positive set . (**B**) PPI information between the IDs in  is assumed unknown. (**C**) The gene IDs in  are the input for Bio::Homology::InterologWalk. (**D**) The putative PPI set obtained is compared with the experimental interaction known positive set.

We tested the functionality of the Bio::Homology::InterologWalk package by recovering known interactions using the orthologue walking principle (Figure 3). To identify known interologs for the validation analyses, we obtained the complete genomes for five well-annotated species (human, mouse, yeast, fly and worm) from Ensembl V. 61. Then, we extracted all the known experimental protein-protein associations for each of the five genomes *G_i _*(*i *= 1, ..., 5) from EBI-Intact. We define  to be the set of the  experimental protein-protein interaction pairs in *G_i_*:

Next, we selected five pairwise genome combinations *G_i_G_j_*: mouse-human, human-yeast, human-fly, fly-yeast and yeast-worm. For each *G_i_G_j_*, we define the *Known Positive Evidence *dataset  as the following subset of :(6)

where ortho(*·*) is the orthology operator.  is the set of all binary PPIs in *G_i _*that match through orthology in *G_j _*(Figure 3A).

**Figure 4:**
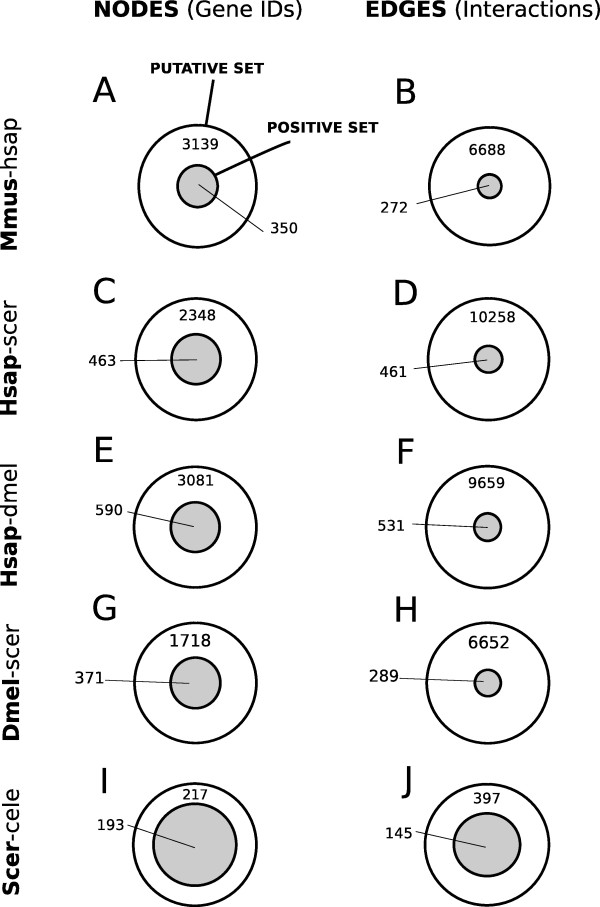
**Known Positive Set and Algorithm Prediction Overlap**. Venn diagrams showing, for five representative species-pair combinations, the overlap between known positive sets  (grey circle) and Bio::Homology::InterologWalk predicted set (white circle). In all observed cases, the algorithm completely rescues the known positive samples and, in addition, proposes new potential interactions and interaction candidates. The new predictions account for a minimum of 53% to a maximum of 90% of the total IDs produced and a minimum of 73% to a maximum of 96% of the total PPIs. The results suggest that even in the case of well studied organisms - provided that the hypothesis of functional conservation between orthologues is correct - most physical protein associations are still unknown.

The gene IDs in the five PPI sets in  were used as input for the module. To validate the ability of Bio::Homology::InterologWalk to recover known interologs (Figure 3B-D), we compared the degree of overlap between predicted nodes (gene IDs) and edges (PPIs) and known positive nodes and edges, for each of the five sets (Figure 4). For each Venn diagram, the grey set represents the known positive set , while the white set corresponds to the algorithm's predictions.

Bio::Homology::InterologWalk successfully retrieves 100% of the positive PPIs in all cases considered. In addition, putative PPI predictions are retrieved, which are candidates that can be prioritised for experimental evaluation. Interestingly, the known positive sets appear smaller than might be expected between closely related organisms like human and mouse. This might be due to a combination of factors such as (1) the parameters for orthology classification used by Ensembl are very stringent, (2) there are biases in experimental research across organisms (the bulk of experimental predictions in each of the two species might come from experiments in different cellular domain and sub-systems) (3) experimental PPI data will likely contain false positive interactions, which will not map through orthology.

It is also interesting to note that in the case of the yeast-worm pair (Figure 4I and 4J) the number of novel IDs and novel PPIs retrieved is one order of magnitude smaller than in the other four cases. This is consistent with the relatively limited amount of experimental PPI data available for *C. elegans*.

#### Assessing the IPX using Receiver Operating Curves (ROC)

**Figure 5:**
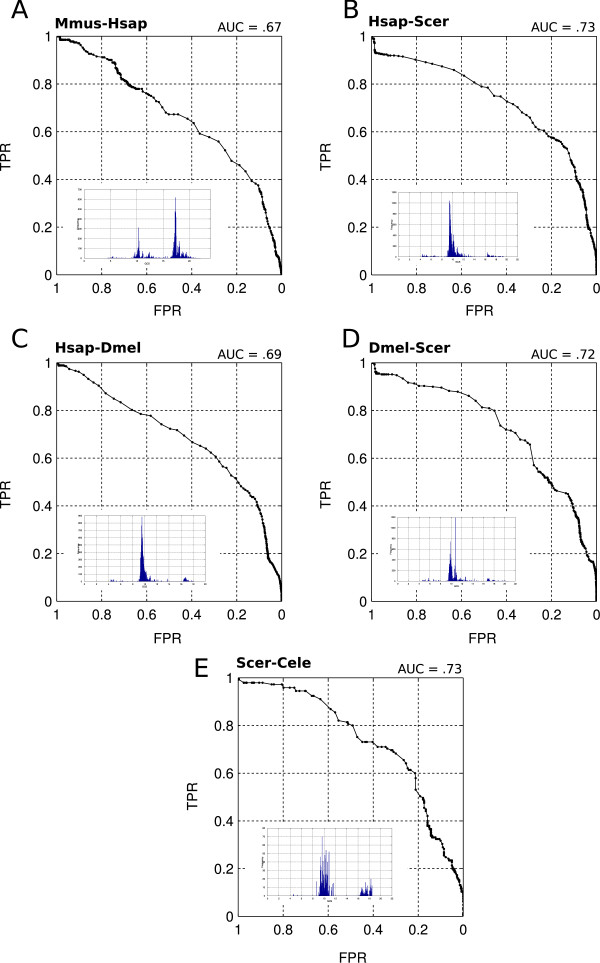
**ROC and IPX score distributions**. Mirrored ROC curves for the five genome pairs in the known positive sets in **KP**. *Inset*: IPX score distributions (reproduced in Additional File 3: 'Interolog Prioritisation Index Histograms'). For each characteristic, the point at coordinate (1,1) corresponds to IPX_thr _= min(IPX), TPR = 100% and FPR = 100%. The point at coordinate (0,0) corresponds to IPX_thr _= max(IPX), TPR = 0% and FPR = 0%. Initially, IPX_thr _= min(IPX). Then, the score histogram is divided into 1000 segments and IPX_thr _is incremented until IPX_thr _= max(IPX) is reached. For all datasets, the decrease of TPR is slower than the decrease of FPR as IPX_thr _→ max(IPX). This means that, as the score threshold becomes more stringent, for all datasets the number of known positive samples lost stays smaller than the number of new predictions lost. The correlation between TPR and the FPR varies depending on the dataset: in the case of the Yeast-Worm pair, 98% of known positives are retrieved when the novel prediction retrieval rate is down to about 76%. Conversely, in the Human-Yeast case, the TPR is down to about 92% for 98% FPR.

Using the known positive datasets in **KP **from the previous section we next calculated ROC curves to assess the performance of the IPX for each of the five species pairs. For all five datasets, the area under the curve AUC *>*0.5 (Figure 5), demonstrating that there is a positive relationship between known positives and the IPX. It is important to note, however, that this correlation varies depending on the dataset. The reason for this is likely to be differences in the completeness of the known positive sets. For all five datasets, the 'real' positive sets are unknown and the disparity between genome size and the number of known positives means that they are likely to represent a small proportion of the 'real' positive set. As a consequence, the AUC values are likely to underestimate the retrieval capability of the algorithm. This also suggests that the IPX may not be optimised. We anticipate that as coverage and categorisation of protein-protein interaction data becomes available it will be possible to optimise the IPX, improve these AUC values and the utility of the metric.

**Figure 6:**
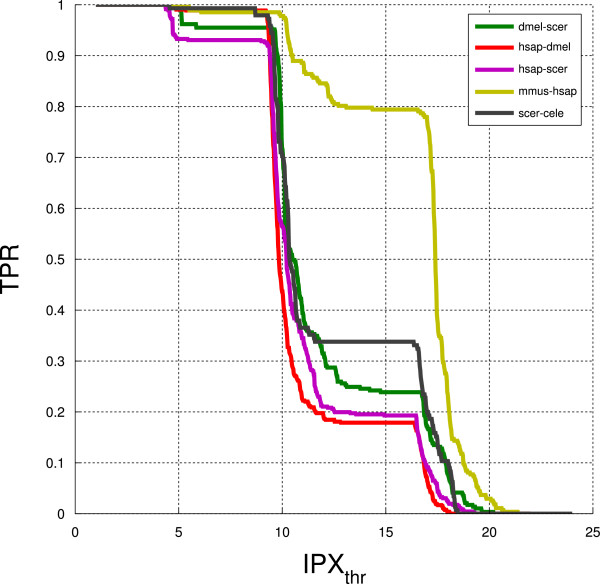
**TPR/IPX Threshold Curve**. Relationship between IPX threshold and known positive sample loss for the five sample datasets. The mouse-human dataset preserves 80% of the positives with a IPX_thr _= 15. At the same threshold value, all the other studied datasets fare significantly worse (Fisher Exact Test, Additional File 2: 'Definitions', Tables S2 and S3).

The reason why a number of known positives have a low index lies in the nature of the IPX. It is designed to reward functionally conserved interologs obtained from binary experimental PPIs. As stated above, the IPX penalises putative PPIs that are from orthology projections where co-orthologues exist or from binary interactions that have been artificially extracted from protein complexes. Some known positives will fall into one or both of these two categories. Additional File 3: 'Interolog Prioritisation Index Histograms' shows IPX distributions for the five known positive datasets in **KP**. Additionally, we show the distribution of the known positives within the IPX histograms in Additional File 4: 'Distribution of positive samples within the IPX histograms'. This chart shows, for each dataset, how many positive samples are in the low (dark), average (medium) and high (bright) tiers of the IPX distribution. For all but the mouse-human genome pair, most known positives fall in the second tier, and the mouse-human dataset is the only one to have most of its positives in the high tier. We examined the relationship between the IPX and the loss of known positives for the five sample datasets (Figure 6 and Additional File 5: 'TPR, FPR and IPX Threshold'). The mouse-human dataset preserves 80% of the positives with an IPX_thr _= 15. At the same threshold value, all of the other datasets fare significantly worse (Fisher Exact Test, Additional File 2: 'Definitions', Tables S2 and S3). These results reflect the closer phylogenetic distance between mouse and human, in which less gene duplication will have occurred since divergence from their common ancestor in comparison to the other species pairs.

It is important to stress that the IPX is not a necessary and sufficient condition to assess the reliability of a putative PPI: a highly ranking interolog may not turn out to be an interesting candidate, but as the index is calculated using both experimental and phylogenetic measures, it would be logical to assess such interologs as candidates before those with lower values. As is the case with any biological scoring metric, a feature with a low score may turn out to be a good candidate and should not be excluded solely on the basis of the score alone.

### Example — Exploring the protein interactome of Drosophila melanogaster

To demonstrate the use of Bio::Homology::InterologWalk, we retrieved a list of all *Drosophila melanogaster *genes (DS_DMEL) from Ensembl-Compara Release 61. For the interolog walk the reference genomes were not restricted to any specific species, but included all 53 available taxa even though we expected a proportion of the species in the sets to provide 'dead end' orthologues where no significant experimental PPI data existed.

For the interolog walk we filtered by retrieving only one-to-one orthologues in the orthology mapping phases and discarded (a) all interactions that were inferred from complexes (spoke) and (b) all interactions that were not experimental physical associations. As a reference we also performed a direct mine of known interactions for the starting gene list with the same interactions filters.

Table 1 shows statistics for the resulting datasets. We adopt the following terminology:

**Table 1:** DS_DMEL - Data for putative and known networks obtained with Bio::Homology::InterologWalk

	DS_DMEL **Pipeline**	
	
	Putative	Known
**Datasets**		
**Gene IDs**	**14869**	**14869**
Reference Genomes	52	NA
Orthologues (Forward)	150968	NA
PPIs in Reference Genomes	37931	NA
Total Interactions	11316	51827
**Unique PP Pairs**	**4428**	**26622**
		
Surviving IDs (% **Gene IDs**)	2188 (14.7)	7779 (52.3)

**Networks**		
**Nodes**	**2188**	**7779**
**Edges**	**4428**	**26622**

The table shows a comparison of the PPI datasets and networks obtained for the *Drosophila melanogaster *interactome dataset (DS_DMEL). Results obtained using the two available

Bio::Homology::InterologWalk pipelines - putative and experimental - are shown. In the putative pipeline, the data shown are relative to interactions obtained through interolog mapping. In the experimental pipeline, the initial dataset has been queried against EBI Intact to gather all known, experimental molecular associations available. The field 'Total Interactions' indicates the total number of final entries of the form *I *= (gene*_x_*, gene*_y_*) obtained. Since *I *can be observed several times through different orthology paths, the field Unique PP Pairs shows the number of unique (gene*_x_*, gene*_y_*) pairs observed.

1. NET_DS_DMEL_known (7779 nodes, 26622 edges) - the network consisting of all the experimentally-obtained physical associations involving genes in DS_DMEL, according to EBI-Intact;

2. NET_DS_DMEL_putative (2188 nodes, 4428 edges) - the network consisting of putative interactions involving genes in DS_DMEL according to Bio::Homology::InterologWalk (filtered as described above);

3. NET_DS_DMEL_union (8270 nodes, 31050 edges) - the network obtained computing the union of (1) and (2) where:

• each node is a node of NET_DS_DMEL_known, NET_DS_DMEL_putative, or both;

• each edge is either an edge of NET_DS_DMEL_known or an edge of NET_DS_DMEL_putative (Note: duplicate edges were *not *collapsed into one).

**Figure 7:**
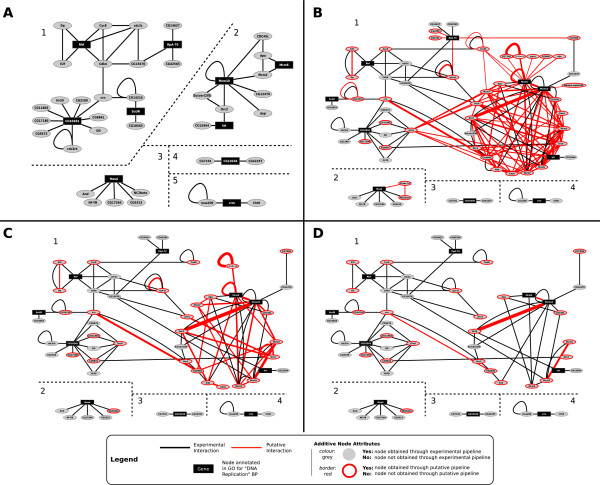
**'DNA Replication' sub-network in **** NET_DS_DMEL_known ****and **** NET_DS_DMEL_union**. **A**: Data extracted from NET_DS_DMEL_known as follows: 1. select 10 core genes annotated with DNA replication GO biological process. 2. select all their nearest neighbours. **B**: NET_DNArep. Obtained as before, but data are extracted from from NET_DS_DMEL_union. **B**-**D**: Using the IPX to refine the putative sub-network in B. We set  (**C**) and  (**D**). IPX score values are mapped to edge thickness in **B**-**D**. Figure 7D shows the sub-network backbone possessing the highest confidence according to the algorithm. Black nodes are genes annotated with the DNA Replication GO biological process. Black connections are experimental PPI data from EBI Intact while red connections are putative predictions taken from NET_DS_DMEL_putative. Nodes are described in key. A high-resolution version of **B **is presented in Additional File 6: 'NET_DNARep'.

In order to explore the results of the interolog walk we analysed the networks using the network tool Cytoscape [39]. Due to the size and complexity of the genome scale interaction networks we decided for the purposes of this illustration to focus on the 65 nodes in NET_DS_DMEL_known that were annotated with the term 'DNA replication' in the Gene Ontology [55]. In order to allow clear visualisation of the data, we further restricted this to a subset of 10 randomly selected genes. We then retrieved all of their nearest neighbours in NET_DS_DMEL_known which produced five disconnected networks (Figure 7A, 46 nodes and 53 edges in total) the biggest of which features 4 DNA replication genes (Figure 7A-1).

To illustrate the utility of the interolog walk we performed the same procedure as above using NET_DS_DMEL_union. In this case we retrieved a set of 68 'DNA replication' genes, a superset of the 65 found before, meaning that 3 additional DNA replication genes are drawn in through the putative pipeline. As before, we selected the sub-network of NET_DS_DMEL_union composed of the 10 core DNA replication genes and their nearest neighbours. The resulting sub-network, NET_DNArep, composed of 68 nodes and 165 edges (Figure 7B and for clarity in higher resolution see Additional File 6: 'NET_DNArep') has greatly increased connectivity (compare Figure 7A to 7B). Indeed the main connected component in NET_DNArep now comprises 55 genes and 153 interactions, and wires together 7 of the 10 core DNA replication genes. A broad analysis of NET_DNArep reveals that the introduction of putative PPI data has allowed us to:

1. collect evidence about new genes, not known to be related to DNA replication before;

2. increase the connectivity of the GO-annotated DNA replication proteins.

Proteins that were known to be involved in DNA replication now interact with proteins for which no evidence for DNA replication involvement existed, meaning new potential candidates are drawn in to build a more complete picture of the domain.

### Using the IPX to refine the Sub-Network

Given the relatively high number of interactions and participating genes in NET_DNArep, we carried out a refinement of the interaction candidates obtained, using the IPX. As described earlier, Bio::Homology::InterologWalk can optionally calculate a prioritisation index for each of the putative PPIs produced. These can be employed to define a 'core' network for which there is strong biological and experimental evidence by removing nodes that are connected by putative PPIs with low IPX indices.

In order to look at the composition of putative PPIs in NET_DNArep, we set ,  and mapped score values to edge thickness in NET_DNArep (Figure 7B-D). Figure 7D shows the sub-network backbone possessing the highest confidence according to the algorithm. Interestingly, the connectedness of the main component still remains at this threshold level. Four putative PPIs survive the strict threshold: (Orc2, Mcm10), (MCM5, MCM3), (MCM10, MCM6) and (crn, CG6905). All these pairs, apart from (MCM6, MCM10), are known to interact experimentally (black edge) and the putative interaction (red edge) reconfirms these results. The (MCM6, MCM10) pair, on the other hand, has not been shown to interact in *Drosophila melanogaster *and represents a candidate for lab validation.

## Conclusions

In this paper, we present Bio::Homology::InterologWalk, a Perl module to retrieve, prioritise and visualise putative Protein-Protein Interactions using interolog mapping. Unlike previous efforts, this Perl library (a) automatically connects to orthology/PPI data web-services to generate up-to-date predictions 'on the fly'. (b) outputs its predictions in the form of simple text files, allowing to use its methods, or the data it produces, within the context of pipeline-based work flows of wider scope (c) optionally flags the predictions on the basis of related biological metadata through a prioritisation index, allowing the selection of a subset of candidates for *in vivo *validation.

We formally validate the efficacy of the tool and present ROC curves to assess the association between the IPX and 'true-positive' interactions across several inter-species 'true-positive' sets. We test the potential of the tool to retrieve putative PPIs on the *Drosophila melanogaster *genome and look more closely at one DNA replication related sub-network identifying several novel nodes and interactions. We conclude by using IPX thresholds to create a 'core' network for which there is strong biological and experimental support.

Our interaction prioritisation index (IPX) is designed to encapsulate biologically relevant principles that relate directly to the assessments currently made manually by many researchers using interaction data. We stress, however, that the IPX measure for an interaction is not fully explored here and that a full validation is not possible due to the current poor coverage of PPI data across species. In our experience the IPX has proven to be a useful summary of biological metadata for PPIs. When performing an interolog walk we recommend the user first uses filtering options to restrict the interactions retrieved and then uses the IPX as a pragmatic aid to candidate prioritisation.

Bio::Homology::InterologWalk is freely available for non-commercial purposes on the Comprehensive Perl Archive Network (CPAN) and modifiable under the GNU GPL license. The package includes full documentation and example scripts to simplify usage.

## Availability and requirements

**Project name **: Bio::Homology::InterologWalk

**Project home page **: http://search.cpan.org/~ggallone/Bio-Homology-InterologWalk/

**Programming Language **: Perl

**Other requirements **: Bioperl, Ensembl API. The module also relies on a number of pre-requisite Perl libraries. See manual on website.

**License **: GNU GPL

## List of abbreviations

**API**: Application Programming Interface; **COG**: Cluster Of Orthologs; **CPAN**: Comprehensive Perl Archive Network; **EBI**: European Bioinformatics Institute; **IPX**: Interolog Prioritisation indeX; **GO**: Gene Ontology; **HUPO-PSI**: HUman Proteome Organisation - Proteomics Standards Initiative; **MITAB**: Molecular Interactions TAB delimited data exchange format; **PCS**: PPI Conservation Score; **PPI**: Protein-Protein Interaction; **PSICQUIC**: Protemics Standard Initiative Common QUery InterfaCe; **PSI-MI**: Proteomics Standard Initiative - Molecular Interactions; **REST**: Representational State Transfer; **ROC**: Receiver Operating Characteristic; **TAP**: Tandem Affinity Purification; **TreeBeST**: (gene) Tree Building guided by Species Tree.

## Competing interests

The authors declare no competing interests.

## Authors' contributions

GG designed and wrote the Bio::Homology::InterologWalk module and performed all of the analyses. APJ, TIS and JDA supervised the work, and all authors contributed to the preparation of the final manuscript.

## Supplementary Material

Additional file 1 **Simplified schematics of the Interolog Walk pipelines**. Flow Diagram documenting the structure and data sources on which the Bio::Homology::InterologWalk pipeline implementation is based. Click here for file

Additional file 2 **Definitions**. Supplementary text providing Bio::Homology::InterologWalk implementation details, design decisions and mathematical background. Click here for file

Additional file 3 **Interolog Prioritisation Index Histograms**. IPX Histograms for the five putative PPI datasets built from the Positive datasets. Click here for file

Additional file 4 **Distribution of positive samples within the IPX histograms**. Distribution of known positive samples in the IPX histograms. The chart shows, for each of the datasets in **KP**, the number of known positive samples in the low (dark), average (medium) and high (bright) tiers of the IPX distribution. Click here for file

Additional file 5 **TPR, FPR and IPX Threshold**. Relationship between TPR, FPR and IPX Threshold for the five putative PPI datasets obtained from the Positive datasets through Bio::Homology::InterologWalk. Click here for file

Additional file 6 **Putative sub-network based on 10 core DNA Replication genes**. Click here for file

## References

[B1] Bray D Molecular Networks: The Top-Down View Science 2003 301 5641 1864 1865 http://www.sciencemag.org/cgi/content/abstract/301/5641/1864 10.1126/science.1089118 14512614

[B2] Giot L Bader JS Brouwer C Chaudhuri A Kuang B Li Y Hao YL Ooi CE Godwin B Vitols E Vijayadamodar G Pochart P Machineni H Welsh M Kong Y Zerhusen B Malcolm R Varrone Z Collis A Minto M Burgess S McDaniel L Stimpson E Spriggs F Williams J Neurath K Ioime N Agee M Voss E Furtak K Renzulli R Aanensen N Carrolla S Bickelhaupt E Lazovatsky Y DaSilva A Zhong J Stanyon CA Finley JRL White KP Braverman M Jarvie T Gold S Leach M Knight J Shimkets RA McKenna MP Chant J Rothberg JM A Protein Interaction Map of Drosophila melanogaster Science 2003 302 5651 1727 1736 http://www.sciencemag.org/cgi/content/abstract/302/5651/1727 10.1126/science.1090289 14605208

[B3] Li S Armstrong CM Bertin N Ge H Milstein S Boxem M Vidalain PO Han JDJ Chesneau A Hao T Goldberg DS Li N Martinez M Rual JF Lamesch P Xu L Tewari M Wong SL Zhang LV Berriz GF Jacotot L Vaglio P Reboul J Hirozane-Kishikawa T Li Q Gabel HW Elewa A Baumgartner B Rose DJ Yu H Bosak S Sequerra R Fraser A Mango SE Saxton WM Strome S van den Heuvel S Piano F Vandenhaute J Sardet C Gerstein M Doucette-Stamm L Gunsalus KC Harper JW Cusick ME Roth FP Hill DE Vidal M A Map of the Interactome Network of the Metazoan C. elegans Science 2004 303 5657 540 543 http://www.sciencemag.org/cgi/content/abstract/303/5657/540 10.1126/science.1091403 14704431 PMC1698949

[B4] LaCount DJ Vignali M Chettier R Phansalkar A Bell R Hesselberth JR Schoenfeld LW Ota I Sahasrabudhe S Kurschner C Fields S Hughes RE A protein interaction network of the malaria parasite Plasmodium falciparum Nature 2005 438 7064 103 107 10.1038/nature04104 16267556

[B5] Stelzl U Worm U Lalowski M Haenig C Brembeck FH Goehler H Stroedicke M Zenkner M Schoenherr A Koeppen S Timm J Mintzlaff S Abraham C Bock N Kietzmann S Goedde A Toksöz E Droege A Krobitsch S Korn B Birchmeier W Lehrach H Wanker EE A Human Protein-Protein Interaction Network: A Resource for Annotating the Proteome Cell 2005 122 6 957 968 http://www.sciencedirect.com/science/article/B6WSN-4H3YGBS-1/2/d39ee6e848fc3d640dcccc8f9ce59eaf 10.1016/j.cell.2005.08.029 16169070

[B6] Yu H Braun P Yildirim MA Lemmens I Venkatesan K Sahalie J Hirozane-Kishikawa T Gebreab F Li N Simonis N Hao T Rual JF Dricot A Vazquez A Murray RR Simon C Tardivo L Tam S Svrzikapa N Fan C de Smet AS Motyl A Hudson ME Park J Xin X Cusick ME Moore T Boone C Snyder M Roth FP Barabasi AL Tavernier J Hill DE Vidal M High-Quality Binary Protein Interaction Map of the Yeast Interactome Network Science 2008 322 5898 104 110 http://www.sciencemag.org/cgi/content/abstract/322/5898/104 10.1126/science.1158684 18719252 PMC2746753

[B7] Figeys D McBroom LD Moran MF Mass Spectrometry for the Study of Protein-Protein Interactions Methods 2001 24 3 230 239 http://www.sciencedirect.com/science/article/B6WN5-456JRHJ-18/2/6dc34bdb705478365b17cb5362d13b56 10.1006/meth.2001.1184 11403572

[B8] Krogan NJ Cagney G Yu H Zhong G Guo X Ignatchenko A Li J Pu S Datta N Tikuisis AP Punna T Peregrín-Alvarez JM Shales M Zhang X Davey M Robinson MD Paccanaro A Bray JE Sheung A Beattie B Richards DP Canadien V Lalev A Mena F Wong P Starostine A Canete MM Vlasblom J Wu S Orsi C Collins SR Chandran S Haw R Rilstone JJ Gandi K Thompson NJ Musso G St Onge P Ghanny S Lam MHY Butland G Altaf-Ul AM Kanaya S Shilatifard A O'Shea E Weissman JS Ingles CJ Hughes TR Parkinson J Gerstein M Wodak SJ Emili A Greenblatt JF Global landscape of protein complexes in the yeast Saccharomyces cerevisiae Nature 2006 440 7084 637 643 10.1038/nature04670 16554755

[B9] Ewing RM Chu P Elisma F Li H Taylor P Climie S McBroom-Cerajewski L Robinson MD O'Connor L Li M Taylor R Dharsee M Ho Y Heilbut A Moore L Zhang S Ornatsky O Bukhman YV Ethier M Sheng Y Vasilescu J Abu-Farha M Lambert JP Duewel HS Stewart II Kuehl B Hogue K Colwill K Gladwish K Muskat B Kinach R Adams SL Moran MF Morin GB Topaloglou T Figeys D Large-scale mapping of human protein-protein interactions by mass spectrometry Mol Syst Biol 2007 3 10.1038/msb4100134 PMC1847948 17353931

[B10] Valencia A Pazos F Computational methods for the prediction of protein interactions Current Opinion in Structural Biology 2002 12 3 368 373 http://www.sciencedirect.com/science/article/B6VS6-469GK4C-J/2/9c30d085fb9e074bbf1d33355fd723f5 10.1016/S0959-440X(02)00333-0 12127457

[B11] Berggård T Linse S James P Methods for the detection and analysis of protein-protein interactions Proteomics 2007 7 16 2833 2842 10.1002/pmic.200700131 17640003

[B12] Bork P Dandekar T Diaz-Lazcoz Y Eisenhaber F Huynen M Yuan Y Predicting function: from genes to genomes and back Journal of Molecular Biology 1998 283 4 707 725 http://www.sciencedirect.com/science/article/B6WK7-45S492P-35/2/10490c37ccfb97ae7e78adebce867c5a 10.1006/jmbi.1998.2144 9790834

[B13] Hegyi H Gerstein M Annotation Transfer for Genomics: Measuring Functional Divergence in Multi-Domain Proteins Genome Research 2001 11 10 1632 1640 http://genome.cshlp.org/content/11/10/1632.abstract 10.1101/gr.183801 11591640 PMC311165

[B14] Walhout AJ Sordella R Lu X Hartley JL Temple GF Brasch MA Thierry-Mieg N Vidal M Protein Interaction Mapping in C. elegans Using Proteins Involved in Vulval Development Science 2000 287 5450 116 122 http://www.sciencemag.org/cgi/content/abstract/287/5450/116 10.1126/science.287.5450.116 10615043

[B15] Matthews LR Vaglio P Reboul J Ge H Davis BP Garrels J Vincent S Vidal M Identification of Potential Interaction Networks Using Sequence-Based Searches for Conserved Protein-Protein Interactions or "Interologs" Genome Research 2001 11 12 2120 2126 http://genome.cshlp.org/content/11/12/2120.abstract 10.1101/gr.205301 11731503 PMC311221

[B16] Huang TW Tien AC Huang WS Lee YCG Peng CL Tseng HH Kao CY Huang CYF POINT: a database for the prediction of protein-protein interactions based on the orthologous interactome Bioinformatics 2004 20 17 3273 3276 http://bioinformatics.oxfordjournals.org/content/20/17/3273.abstract 10.1093/bioinformatics/bth366 15217821

[B17] Lehner B Fraser A A first-draft human protein-interaction map Genome Biology 2004 5 9 R63 http://genomebiology.com/2004/5/9/R63 10.1186/gb-2004-5-9-r63 15345047 PMC522870

[B18] Brown KR Jurisica I Online Predicted Human Interaction Database Bioinformatics 2005 21 9 2076 2082 http://bioinformatics.oxfordjournals.org/content/21/9/2076.abstract 10.1093/bioinformatics/bti273 15657099

[B19] Persico M Ceol A Gavrila C Hoffmann R Florio A Cesareni G HomoMINT: an inferred human network based on orthology mapping of protein interactions discovered in model organisms BMC Bioinformatics 2005 6 Suppl 4 S21 http://www.biomedcentral.com/1471-2105/6/S4/S21 10.1186/1471-2105-6-S4-S21 16351748 PMC1866386

[B20] Kemmer D Huang Y Shah S Lim J Brumm J Yuen M Ling J Xu T Wasserman W Ouellette BF Ulysses - an application for the projection of molecular interactions across species Genome Biology 2005 6 12 R106 http://genomebiology.com/2005/6/12/R106 10.1186/gb-2005-6-12-r106 16356269 PMC1414088

[B21] Gandhi TKB Zhong J Mathivanan S Karthick L Chandrika KN Mohan SS Sharma S Pinkert S Nagaraju S Periaswamy B Mishra G Nandakumar K Shen B Deshpande N Nayak R Sarker M Boeke JD Parmigiani G Schultz J Bader JS Pandey A Analysis of the human protein interactome and comparison with yeast, worm and fly interaction datasets Nat Genet 2006 38 3 285 293 10.1038/ng1747 16501559

[B22] Huang TW Lin CY Kao CY Reconstruction of human protein interolog network using evolutionary conserved network BMC Bioinformatics 2007 8 152 http://www.biomedcentral.com/1471-2105/8/152 10.1186/1471-2105-8-152 17493278 PMC1885812

[B23] Wojcik J Boneca IG Legrain P Prediction, Assessment and Validation of Protein Interaction Maps in Bacteria Journal of Molecular Biology 2002 323 4 763 770 http://www.sciencedirect.com/science/article/B6WK7-473VN5W-F/2/786fdc31b9e4e8c7bc795c0f423c5859 10.1016/S0022-2836(02)01009-4 12419263

[B24] Sharan R Ideker T Kelley B Shamir R Karp RM Identification of Protein Complexes by Comparative Analysis of Yeast and Bacterial Protein Interaction Data Journal of Computational Biology 2005 12 6 835 846 http://www.liebertonline.com/doi/abs/10.1089/cmb.2005.12.835 10.1089/cmb.2005.12.835 16108720

[B25] Wuchty S Ipsaro JJ A Draft of Protein Interactions in the Malaria Parasite P. falciparum Journal of Proteome Research 2007 6 4 1461 1470 http://pubs.acs.org/doi/abs/10.1021/pr0605769 10.1021/pr0605769 17300188

[B26] He F Zhang Y Chen H Zhang Z Peng YL The prediction of protein-protein interaction networks in rice blast fungus BMC Genomics 2008 9 519 http://www.biomedcentral.com/1471-2164/9/519 10.1186/1471-2164-9-519 18976500 PMC2601049

[B27] Yu H Luscombe NM Lu HX Zhu X Xia Y Han JDJ Bertin N Chung S Vidal M Gerstein M Annotation Transfer Between Genomes: Protein-Protein Interologs and Protein-DNA Regulogs Genome Research 2004 14 6 1107 1118 http://genome.cshlp.org/content/14/6/1107.abstract 10.1101/gr.1774904 15173116 PMC419789

[B28] Michaut M Kerrien S Montecchi-Palazzi L Chauvat F Cassier-Chauvat C Aude JC Legrain P Hermjakob H InteroPORC: automated inference of highly conserved protein interaction networks Bioinformatics 2008 24 14 1625 1631 http://bioinformatics.oxfordjournals.org/content/24/14/1625.abstract 10.1093/bioinformatics/btn249 18508856

[B29] Wiles A Doderer M Ruan J Gu TT Ravi D Blackman B Bishop A Building and analyzing protein interactome networks by cross-species comparisons BMC Systems Biology 2010 4 36 http://www.biomedcentral.com/1752-0509/4/36 10.1186/1752-0509-4-36 20353594 PMC2859380

[B30] Kersey P Bower L Morris L Horne A Petryszak R Kanz C Kanapin A Das U Michoud K Phan I Gattiker A Kulikova T Faruque N Duggan K Mclaren P Reimholz B Duret L Penel S Reuter I Apweiler R Integr8 and Genome Reviews: integrated views of complete genomes and proteomes Nucleic Acids Research 2005 33 suppl 1 D297 D302 http://nar.oxfordjournals.org/content/33/suppl_1/D297.abstract 15608201 10.1093/nar/gki039 PMC539993

[B31] Pedamallu CS Posfai J Open source tool for prediction of genome wide protein-protein interaction network based on ortholog information Source Code for Biology and Medicine 2010 5 8 http://www.scfbm.org/content/5/1/8 10.1186/1751-0473-5-8 20684769 PMC2924336

[B32] Stajich JE Block D Boulez K Brenner SE Chervitz SA Dagdigian C Fuellen G Gilbert JG Korf I Lapp H Lehväslaiho H Matsalla C Mungall CJ Osborne BI Pocock MR Schattner P Senger M Stein LD Stupka E Wilkinson MD Birney E The Bioperl Toolkit: Perl Modules for the Life Sciences Genome Research 2002 12 10 1611 1618 http://genome.cshlp.org/content/12/10/1611.abstract 10.1101/gr.361602 12368254 PMC187536

[B33] Kersey PJ Lawson D Birney E Derwent PS Haimel M Herrero J Keenan S Kerhornou A Koscielny G Kähäri A Kinsella RJ Kulesha E Maheswari U Megy K Nuhn M Proctor G Staines D Valentin F Vilella AJ Yates A Ensembl Genomes: Extending Ensembl across the taxonomic space Nucleic Acids Research 2010 38 suppl 1 D563 D569 http://nar.oxfordjournals.org/content/38/suppl_1/D563.abstract 19884133 10.1093/nar/gkp871 PMC2808935

[B34] Flicek P Aken BL Ballester B Beal K Bragin E Brent S Chen Y Clapham P Coates G Fairley S Fitzgerald S Fernandez-Banet J Gordon L Gräf S Haider S Hammond M Howe K Jenkinson A Johnson N Kähäri A Keefe D Keenan S Kinsella R Kokocinski F Koscielny G Kulesha E Lawson D Longden I Massingham T McLaren W Megy K Overduin B Pritchard B Rios D Ruffier M Schuster M Slater G Smedley D Spudich G Tang YA Trevanion S Vilella A Vogel J White S Wilder SP Zadissa A Birney E Cunningham F Dunham I Durbin R Fern_andez-Suarez XM Herrero J Hubbard TJP Parker A Proctor G Smith J Searle SMJ Ensembl's 10th year Nucleic Acids Research 2010 38 suppl 1 D557 D562 http://nar.oxfordjournals.org/content/38/suppl_1/D557.abstract 19906699 10.1093/nar/gkp972 PMC2808936

[B35] Kerrien S Orchard S Montecchi-Palazzi L Aranda B Quinn A Vinod N Bader G Xenarios I Wojcik J Sherman D Tyers M Salama J Moore S Ceol A Chatr-aryamontri A Oesterheld M Stumpen V Salwinski L Nerothin J Cerami E Cusick M Vidal M Gilson M Armstrong J Woollard P Hogue C Eisenberg D Cesareni G Apweiler R Hermjakob H Broadening the horizon - level 2.5 of the HUPO-PSI format for molecular interactions BMC Biology 2007 5 44 http://www.biomedcentral.com/1741-7007/5/44 10.1186/1741-7007-5-44 17925023 PMC2189715

[B36] Aranda B Achuthan P Alam-Faruque Y Armean I Bridge A Derow C Feuermann M Ghanbarian AT Kerrien S Khadake J Kerssemakers J Leroy C Menden M Michaut M Montecchi-Palazzi L Neuhauser SN Orchard S Perreau V Roechert B van Eijk K Hermjakob H The IntAct molecular interaction database in 2010 Nucleic Acids Research 2010 38 suppl 1 D525 D531 http://nar.oxfordjournals.org/content/38/suppl_1/D525.abstract 19850723 10.1093/nar/gkp878 PMC2808934

[B37] The Comprehensive Perl Archive Network http://www.cpan.org/

[B38] Gallone G Bio::Homology::InterologWalk - Retrieve, score and visualize putative Protein-Protein Interactions through the orthology-walk method 2011 http://search.cpan.org/~ggallone/Bio-Homology-InterologWalk/

[B39] Shannon P Markiel A Ozier O Baliga NS Wang JT Ramage D Amin N Schwikowski B Ideker T Cytoscape: A Software Environment for Integrated Models of Biomolecular Interaction Networks Genome Research 2003 13 11 2498 2504 http://genome.cshlp.org/content/13/11/2498.abstract 10.1101/gr.1239303 14597658 PMC403769

[B40] Adams MD Celniker SE Holt RA Evans CA Gocayne JD Amanatides PG Scherer SE Li PW Hoskins RA Galle RF George RA Lewis SE Richards S Ashburner M Henderson SN Sutton GG Wortman JR Yandell MD Zhang Q Chen LX Brandon RC Rogers YHC Blazej RG Champe M Pfeiffer BD Wan KH Doyle C Baxter EG Helt G Nelson CR Gabor GL Abril JF Agbayani A An HJ Andrews-Pfannkoch C Baldwin D Ballew RM Basu A Baxendale J Bayraktaroglu L Beasley EM Beeson KY Benos PV Berman BP Bhandari D Bolshakov S Borkova D Botchan MR Bouck J Brokstein P Brottier P Burtis KC Busam DA Butler H Cadieu E Center A Chandra I Cherry JM Cawley S Dahlke C Davenport LB Davies P Pablos Bd Delcher A Deng Z Mays AD Dew I Dietz SM Dodson K Doup LE Downes M Dugan-Rocha S Dunkov BC Dunn P Durbin KJ Evangelista CC Ferraz C Ferriera S Fleischmann W Fosler C Gabrielian AE Garg NS Gelbart WM Glasser K Glodek A Gong F Gorrell JH Gu Z Guan P Harris M Harris NL Harvey D Heiman TJ Hernandez JR Houck J Hostin D Houston KA Howland TJ Wei MH Ibegwam C Jalali M Kalush F Karpen GH Ke Z Kennison JA Ketchum KA Kimmel BE Kodira CD Kraft C Kravitz S Kulp D Lai Z Lasko P Lei Y Levitsky AA Li J Li Z Liang Y Lin X Liu X Mattei B McIntosh TC McLeod MP McPherson D Merkulov G Milshina NV Mobarry C Morris J Moshrefi A Mount SM Moy M Murphy B Murphy L Muzny DM Nelson DL Nelson DR Nelson KA Nixon K Nusskern DR Pacleb JM Palazzolo M Pittman GS Pan S Pollard J Puri V Reese MG Reinert K Remington K Saunders RDC Scheeler F Shen H Shue BC Sidén-Kiamos I Simpson M Skupski MP Smith T Spier E Spradling AC Stapleton M Strong R Sun E Svirskas R Tector C Turner R Venter E Wang AH Wang X Wang ZY Wassarman DA Weinstock GM Weissenbach J Williams SM Woodage T Worley KC Wu D Yang S Yao QA Ye J Yeh RF Zaveri JS Zhan M Zhang G Zhao Q Zheng L Zheng XH Zhong FN Zhong W Zhou X Zhu S Zhu X Smith HO Gibbs RA Myers EW Rubin GM Venter JC The Genome Sequence of Drosophila melanogaster Science 2000 287 5461 2185 2195 http://www.sciencemag.org/content/287/5461/2185.abstract 10.1126/science.287.5461.2185 10731132

[B41] Vilella AJ Severin J Ureta-Vidal A Heng L Durbin R Birney E EnsemblCompara GeneTrees: Complete, duplication-aware phylogenetic trees in vertebrates Genome Research 2009 19 2 327 335 http://genome.cshlp.org/content/19/2/327.abstract 19029536 10.1101/gr.073585.107 PMC2652215

[B42] Fielding RT Taylor RN Principled design of the modern Web architecture ICSE '00: Proceedings of the 22nd international conference on Software engineering 2000 New York, NY, USA: ACM 407 416 http://portal.acm.org/citation.cfm?id=337228

[B43] Prieto C De Las Rivas J APID: Agile Protein Interaction DataAnalyzer Nucleic Acids Research 2006 34 suppl 2 W298 W302 http://nar.oxfordjournals.org/content/34/suppl_2/W298.abstract 16845013 10.1093/nar/gkl128 PMC1538863

[B44] Razick S Magklaras G Donaldson I iRefIndex: A consolidated protein interaction database with provenance BMC Bioinformatics 2008 9 405 http://www.biomedcentral.com/1471-2105/9/405 10.1186/1471-2105-9-405 18823568 PMC2573892

[B45] Breitkreutz BJ Stark C Reguly T Boucher L Breitkreutz A Livstone M Oughtred R Lackner DH Bähler J Wood V Dolinski K Tyers M The BioGRID Interaction Database: 2008 update Nucleic Acids Research 2008 36 suppl 1 D637 D640 http://nar.oxfordjournals.org/content/36/suppl_1/D637.abstract 18000002 10.1093/nar/gkm1001 PMC2238873

[B46] Goll J Rajagopala SV Shiau SC Wu H Lamb BT Uetz P MPIDB: the microbial protein interaction database Bioinformatics 2008 24 15 1743 1744 http://bioinformatics.oxfordjournals.org/content/24/15/1743.abstract 10.1093/bioinformatics/btn285 18556668 PMC2638870

[B47] Chautard E Ballut L Thierry-Mieg N Ricard-Blum S MatrixDB, a database focused on extracellular protein-protein and protein-carbohydrate interactions Bioinformatics 2009 25 5 690 691 http://bioinformatics.oxfordjournals.org/content/25/5/690.abstract 10.1093/bioinformatics/btp025 19147664 PMC2647840

[B48] Jensen LJ Kuhn M Stark M Chaffron S Creevey C Muller J Doerks T Julien P Roth A Simonovic M Bork P von Mering C STRING 8--a global view on proteins and their functional interactions in 630 organisms Nucleic Acids Research 2009 37 suppl 1 D412 D416 http://nar.oxfordjournals.org/content/37/suppl_1/D412.abstract 18940858 10.1093/nar/gkn760 PMC2686466

[B49] Matthews L Gopinath G Gillespie M Caudy M Croft D de Bono B Garapati P Hemish J Hermjakob H Jassal B Kanapin A Lewis S Mahajan S May B Schmidt E Vastrik I Wu G Birney E Stein L D'Eustachio P Reactome knowledgebase of human biological pathways and processes Nucleic Acids Research 2009 37 suppl 1 D619 D622 http://nar.oxfordjournals.org/content/37/suppl_1/D619.abstract 18981052 10.1093/nar/gkn863 PMC2686536

[B50] Ceol A Chatr Aryamontri A Licata L Peluso D Briganti L Perfetto L Castagnoli L Cesareni G MINT, the molecular interaction database: 2009 update Nucleic Acids Research 2010 38 suppl 1 D532 D539 http://nar.oxfordjournals.org/content/38/suppl_1/D532.abstract 19897547 10.1093/nar/gkp983 PMC2808973

[B51] He X Zhang J Rapid Subfunctionalization Accompanied by Prolonged and Substantial Neofunctionalization in Duplicate Gene Evolution Genetics 2005 169 2 1157 1164 http://www.genetics.org/cgi/content/abstract/169/2/1157 10.1534/genetics.104.037051 15654095 PMC1449125

[B52] Hittinger CT Carroll SB Gene duplication and the adaptive evolution of a classic genetic switch Nature 2007 449 7163 677 681 10.1038/nature06151 17928853

[B53] Fraser HB Hirsh AE Steinmetz LM Scharfe C Feldman MW Evolutionary Rate in the Protein Interaction Network Science 2002 296 5568 750 752 http://www.sciencemag.org/cgi/content/abstract/296/5568/750 10.1126/science.1068696 11976460

[B54] Wuchty S Oltvai ZN Barabasi AL Evolutionary conservation of motif constituents in the yeast protein interaction network Nat Genet 2003 35 2 176 179 10.1038/ng1242 12973352

[B55] Ashburner M Ball CA Blake JA Botstein D Butler H Cherry JM Davis AP Dolinski K Dwight SS Eppig JT Harris MA Hill DP Issel-Tarver L Kasarskis A Lewis S Matese JC Richardson JE Ringwald M Rubin GM Sherlock G Gene Ontology: tool for the unification of biology Nat Genet 2000 25 25 29 10.1038/75556 10802651 PMC3037419

