# Supplementary figures and images for: Bio::Homology::InterologWalk - A Perl module to build putative protein-protein interaction networks through interolog mapping
Source: BMC Bioinformatics. 2011 Jul 18;12:289. doi: 10.1186/1471-2105-12-289 (PMC3161927; doi:10.1186/1471-2105-12-289)

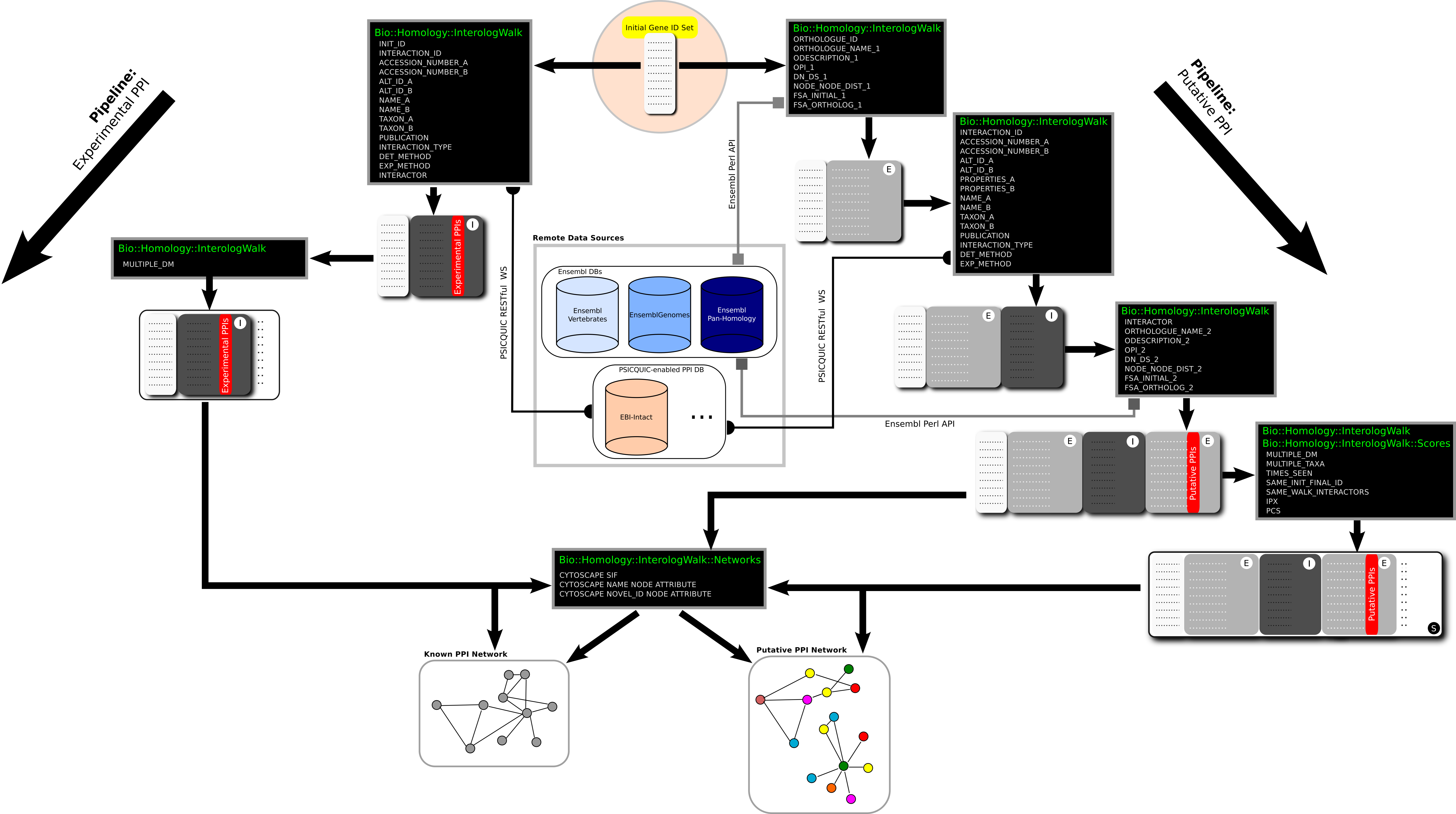

Supplement: Additional file 1 — Simplified schematics of the Interolog Walk pipelines. Flow Diagram documenting the structure and data sources on which the Bio::Homology::InterologWalk pipeline implementation is based. [file 1471-2105-12-289-S1.PDF]

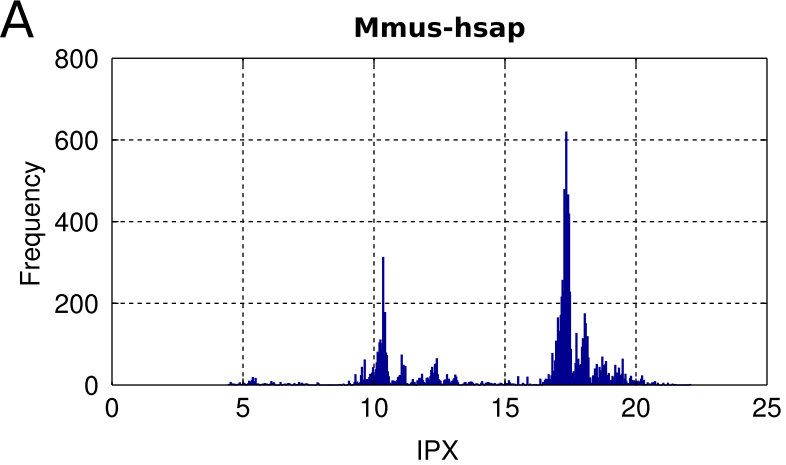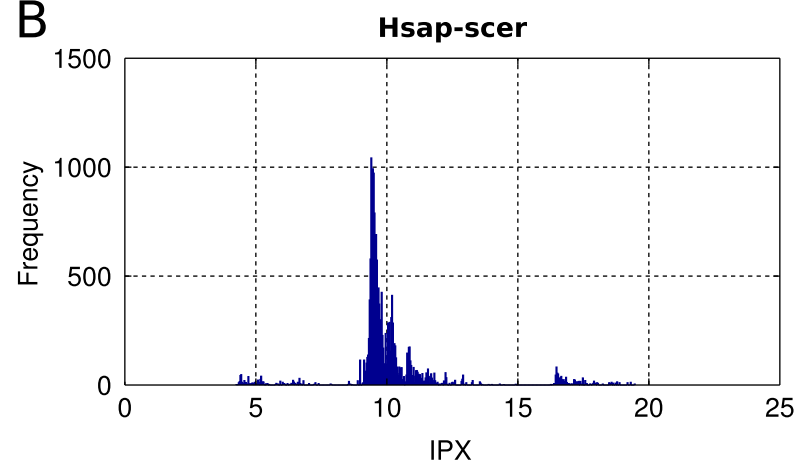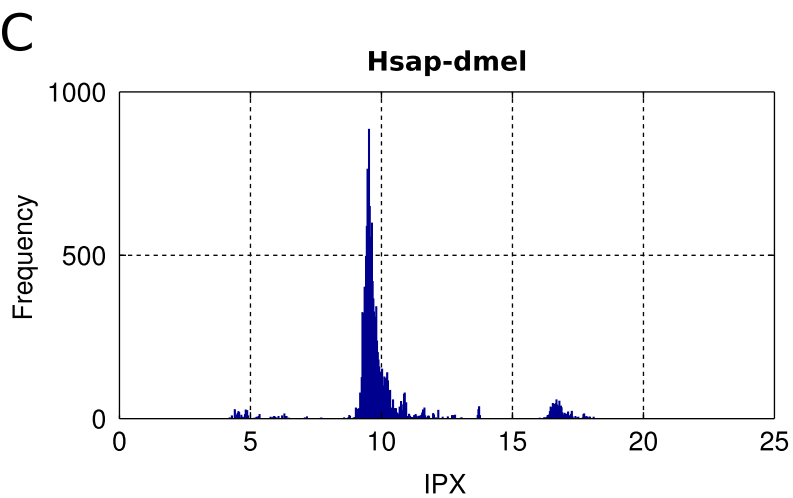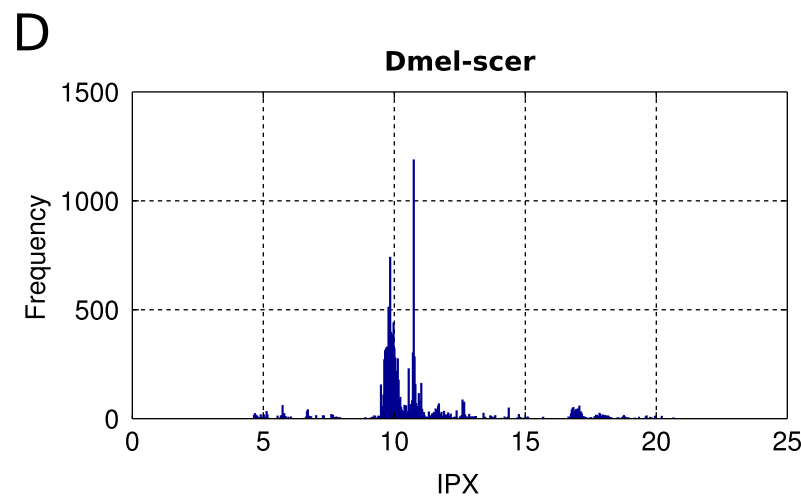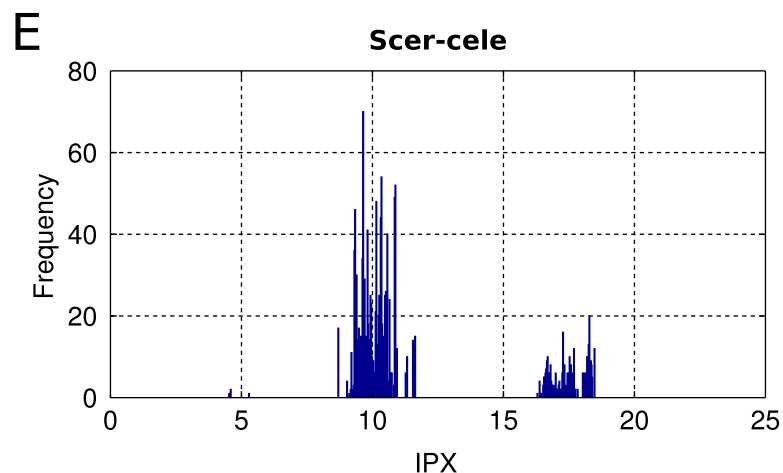

Supplement: Additional file 3 — Interolog Prioritisation Index Histograms. IPX Histograms for the five putative PPI datasets built from the Positive datasets. [file 1471-2105-12-289-S3.PDF]

Distribution of the Known Positive Samples

Number of Known Positive Samples

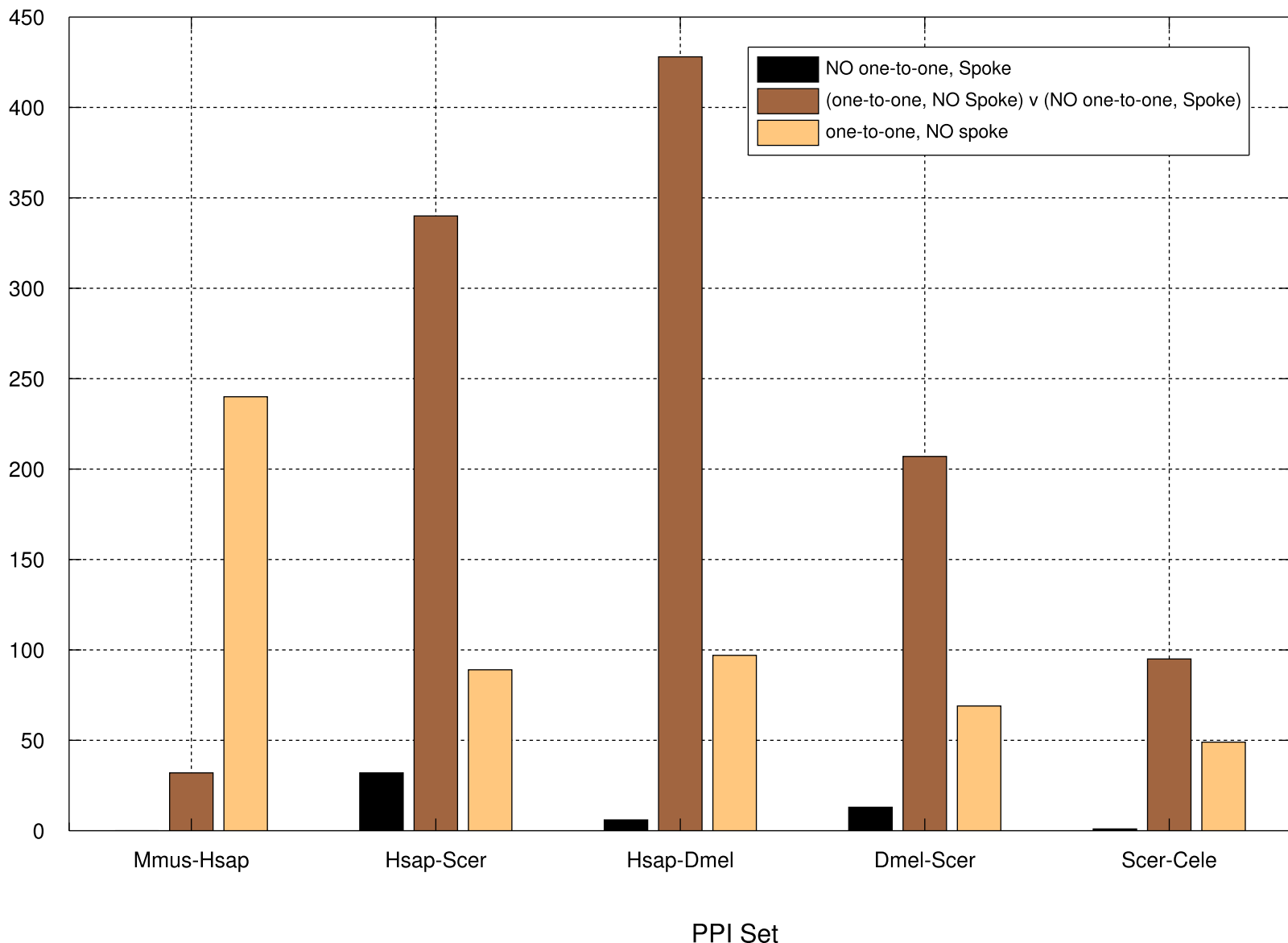

Supplement: Additional file 4 — Distribution of positive samples within the IPX histograms. Distribution of known positive samples in the IPX histograms. The chart shows, for each of the datasets in KP, the number of known positive samples in the low (dark), average (medium) and high (bright) tiers of the IPX distribution. [file 1471-2105-12-289-S4.PDF]

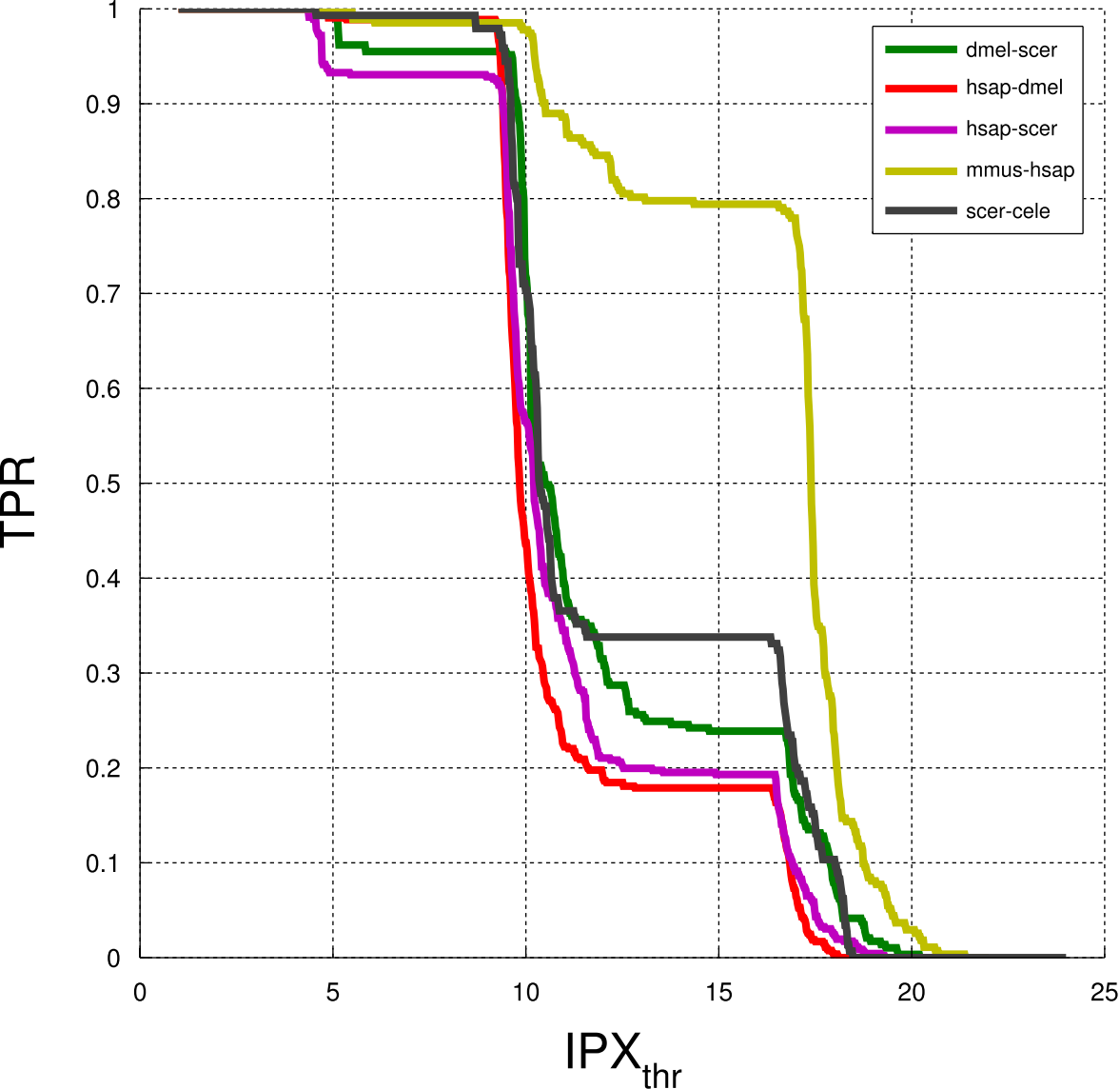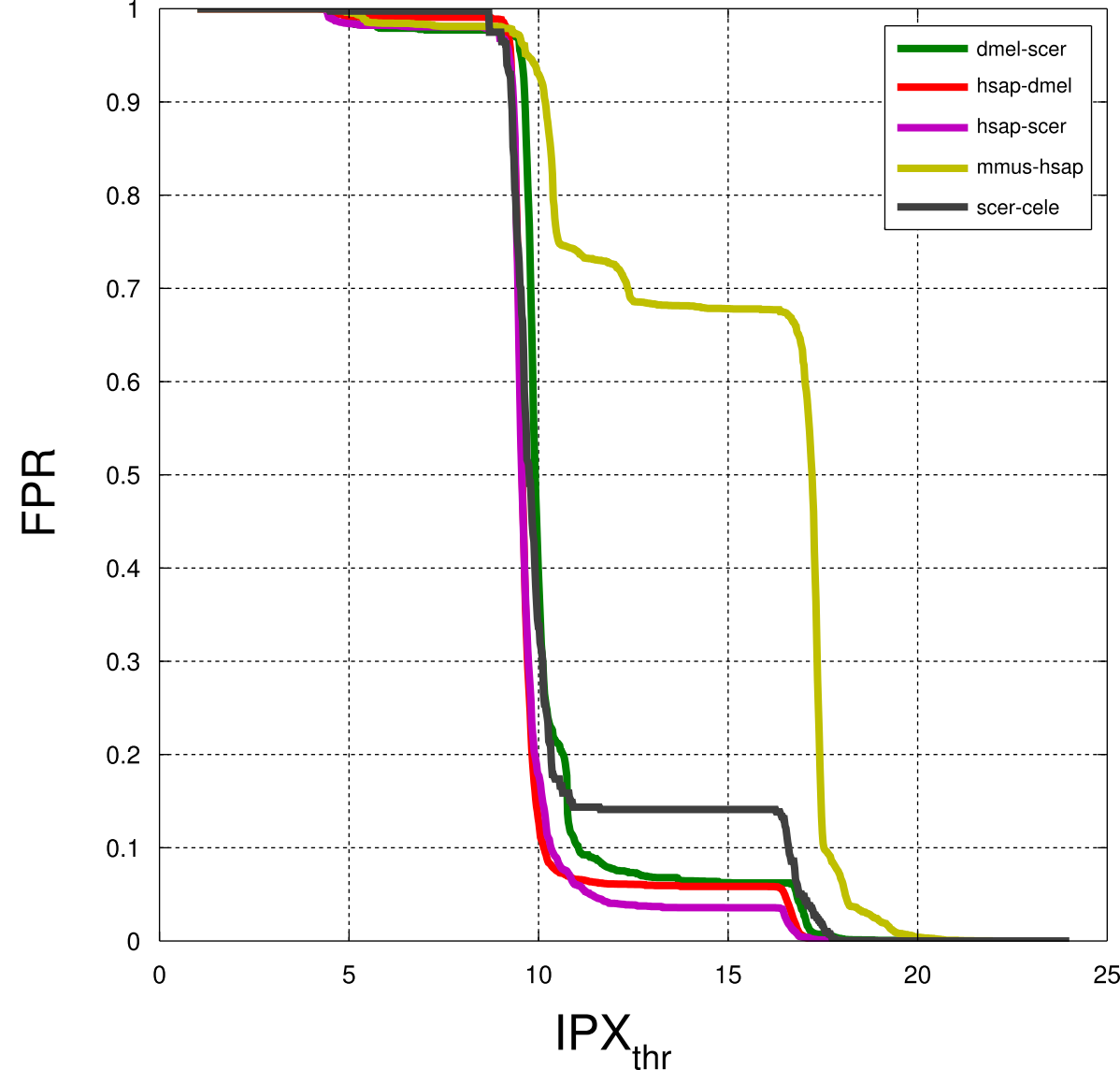

Supplement: Additional file 5 — TPR, FPR and IPX Threshold. Relationship between TPR, FPR and IPX Threshold for the five putative PPI datasets obtained from the Positive datasets through Bio::Homology::InterologWalk. [file 1471-2105-12-289-S5.PDF]
