# Supplementary material for: Bio::Homology::InterologWalk - A Perl module to build putative protein-protein interaction networks through interolog mapping
Source: BMC Bioinformatics. 2011 Jul 18;12:289. doi: 10.1186/1471-2105-12-289 (PMC3161927; doi:10.1186/1471-2105-12-289)
Supplement: Additional file 2 — Definitions. Supplementary text providing Bio::Homology::InterologWalk implementation details, design decisions and mathematical background. [file 1471-2105-12-289-S2.PDF]

Additional file 1 — Definitions

Bio::Homology::InterologWalk – A Perl module to build putative protein-protein interaction networks through interolog mapping

Contents

1 Orthology Predictions from Ensembl Compara **1**
1.1 Ensembl Data Access 2

2 PPI Data from EBI-IntAct **3**
2.1 Gene ID/Protein ID Conversion 3

3 Prioritisation Metrics **4**
3.1 IPX — Interolog Prioritisation Index 4
3.2 PCS — PPI Conservation Score 6

4 Validation **7**
4.1 Fisher Test Data for the Positive Retrieval Analysis 7

1 Orthology Predictions from Ensembl Compara

`Bio::Homology::InterologWalk` relies on the comparative biology data provided by the Ensembl Project.

The orthology prediction method utilised by Ensembl is described in Vilella *et al.* [1] and is based on a computational pipeline having at its core the TreeBeST algorithm (http://treesoft.sourceforge.net/treebest.shtml). TreeBeST is based on a modified version of the Phyml algorithm [2]: given an aligned cluster, it works by reconciling each gene tree with a corresponding species tree.

Ensembl Compara identifies two main types of homology association between genes, *orthologues* and *paralogues*. Two genes in two different species are in an orthology relationship if they derive from a single gene present in their last common ancestor [3]. On the other hand, two genes are called paralogues if they stem from a single gene that underwent duplication within a genome. Given this basic distinction, a number of sub-categories are defined:

Co-orthologues multiple genes in an organism that are simultaneously orthologues of a gene in another organism are termed *co-orthologues* of such gene;

In-Paralogues and Out-Paralogues paralogy can be further distinguished, relative to a speciation event, into an ancestral form, called out-paralogy, and into a recent form, called in-paralogy. Two or more genes are called out-paralogues with respect to a speciation event if the duplication that created them predates the speciation. On the other hand, two or more genes are called in-paralogues relative to a speciation event if the duplication that created them follows the speciation event.

The concepts of in-paralogy and co-orthology are tightly related, and assume a particular significance within the context of our functional transfer study. Specifically, members of a co-orthology group are *in-paralogs* with respect to each other, and relative to the speciation event that created the orthology. Ensembl Compara will classify any gene α, for which at least one orthologue β is found, in one of the following categories:

ortholog one-to-one. α has not undergone duplication (better, according to the TreeBeST algorithm there are no duplicates after the speciation event) This corresponds to saying that α has no in-paralogues. Same for β.

ortholog one-to-many. α has not undergone duplication after the speciation event (α has no in-paralogues). However, β has undergone duplication after the speciation event and has a number of in-paralogues (i.e., β is part of co-orthology group with respect to α).

ortholog many-to-one. α has undergone duplication after the speciation event, and has a number of in-paralogues. α is part of a co-orthology group w.r.t. β and only β.

ortholog many-to-many. Both α and β have undergone duplication after the speciation event. α, with all its in-paralogues, is an orthologue of β and of all its in-paralogues.

Within the context of this study we are particularly interested in *one-to-one* orthologues — orthology relationships where neither of the two members has (according to Compara) undergone duplication after the speciation event. It has been shown that gene duplication is related to neo-functionalization and/or sub-functionalization [4]. With one-to-one orthologues, functional conservation is more likely to be retained [5,6]. Based on the Ensembl annotation, `Bio::Homology::InterologWalk` can act in one of two ways:

- retain putative PPIs in which *both* the orthology projections are of the one-to-one kind only, discarding all other classes;
- keep all putative PPIs, regardless of class, and then optionally use prioritisation metrics to flag the predictions.

Ensembl also identifies cases tagged as "possible ortholog": instances when the duplication/speciation nature of the phylogenetic tree could not be fully resolved by the Compara–TreeBeST algorithm, but partial evidence suggested the absence of a duplication event. As reported by Ensembl, such cases might point to long-distance relations that might be upgraded to *bona fide* orthologies in further versions of the gene tree pipeline.

1.1 Ensembl Data Access

The Ensembl Project offers a wide variety of alternatives in terms of data access. It is possible to query single gene ids using a sophisticated web-interface (accessible via http://www.ensembl.org/), but also to build customized queries of varying complexity using a web tool called BioMart (http://www.ensembl.org/biomart/). These two data-access paradigms are extremely helpful for non-technical users of the data and are useful for quick comparison of data entries: the inherent simplicity of the web-interface paradigm and an extremely versatile advanced graphical user interface make them ideal for such scenarios. However, web-interfaces are not designed for high-throughput, selective mass-retrieval of data entries. As a third alternative, the possibility to download full dumps of the MySQL DBs to set-up off-line data mirrors means that interested researchers can work on large quantities of data without the bottleneck of using a graphical interface.

If downloading a static dump of the data is not —as is the case with the current project — an option, Ensembl offers a very complete framework for programmatic access to the data through their Perl API (http://www.ensembl.org/info/data/api.html). The API serves as a middle-layer between the underlying database schemas (whose complexity is often of no interest to the end user) and more specific application programmes, of which the Perl library we propose, `Bio::Homology::InterologWalk`, is an example.

2 PPI Data from EBI-IntAct

The list of PPI databases that implement the PSICQUIC specification includes APID [7], iRefIndex [8], BioGrid [9], MPIDB [10], InnateDB [11], MatrixDB [12], STRING [13], Reactome [14], EBI IntAct [15], MINT [16] and ChEMBL (http://www.ebi.ac.uk/chembl/). `Bio::Homology::InterologWalk` currently relies on EBI IntAct as its source of experimental interactions. The reason for this choice is manyfold. A number of PSICQUIC-enabled databases are aggregators of interaction data found elsewhere (e.g. APID, MPIDB, MatrixDB, iRefIndex). Some are dedicated to collecting interactions belonging to specialised domains (e.g. InnateDB) while others contain data that is not suited for our purpose (e.g. ChEMBL). The IntAct project [17], hosted at The European Bioinformatics Institute (EBI), offers an open-source, open-data PPI database based on literature data and direct data deposition by expert curators, following a standardised model. As of May 2011, v. 1.1.6 of the IntAct database contains more than 265,000 curated binary protein interaction evidences [15]. All software available on the IntAct site is under the Apache License, version 2, and all data under the Creative Commons Attribution License.

The EBI offers a variety of data access methods. In particular, PSICQUIC [Aranda *et al*, in preparation], a standardized web-service from the HUPO-PSI (http://www.psidev.info/) offers both SOAP- and REST-based data access and a set of common query methods specified in the Molecular Interaction Query Language (MIQL) definition. The data it organized according to the annotation rules defined in the HUPO-PSI Controlled Vocabulary [18]. This standard provides unique designations for meta-data terms like interaction detection method, taxon of the participants, interaction type, interactor type, database, interactor roles, and much more. `Bio::Homology::InterologWalk` queries Intact using the RESTful-based PSICQUIC implementation and retrieves data in PSI-MI MITAB25 tab-delimited format [19]. All protein-protein interaction in IntAct come in the form of binary entries: each data entry is a row identifying a binary PPI interaction and its supporting evidence. One important piece of supplementary information provided by IntAct and processed by `Bio::Homology::InterologWalk` is the *complex expansion* flag. When dealing with the case of complexes of interacting proteins, an assumption has to be made when the decomposition of the complex in binary associations is needed. IntAct adopts a computational ppi-complex expansion paradigm called *Spoke*. In the Spoke model, the experimental results describing the purification of protein complexes are converted into pairwise interactions between bait and preys only. If the complex is composed of n proteins interacting in an unknown configuration, the Spoke model will generate $n-1$ binary associations. An alternative to this is the *fully connected matrix* model, which assumes all proteins in a complex to be connected to all others. Given an n-protein complex, the matrix model will generate $\left[n(n-1)\right]/2$ binary associations.

Both methods have their shortcomings, and will report a certain number of false positives (fully connected matrix) or false negatives *and* false positives (spoke expansion).

2.1 Gene ID/Protein ID Conversion

`Bio::Homology::InterologWalk` retrieves Ensembl Compara orthology data using gene IDs. EBI Intact, however, returns binary PPI information using UniprotKB protein identifiers. In order to return to Ensembl IDs for the backward part of the orthology retrieval, a conversion phase is required and Ensembl IDs must be obtained for each reference experimental interactor. Genes often produce many transcripts via alternative splicing that code for multiple isoforms of the protein. Currently we map all of these isoforms onto the parent gene. This is due to the absence of reliable methods to map isoforms between species, especially when their common ancestor is very distant.

The module will extract Ensembl IDs, when present, from MITAB25 supplementary data fields provided by Intact. If such data is not available, a conversion algorithm will be employed. The IDs in the Intact data entry are generally preferable to avoid the computational burden associated with the ID look-up/conversion algorithm. However, we found that in some cases the gene IDs provided by Intact pointed to obsolete or secondary IDs. As a consequence, `Bio::Homology::InterologWalk` gives the possibility of always double-checking gene ID consistency against Ensembl. We relied on the conversion algorithm for the dataset analysis

we discuss in this paper.

3 Prioritisation Metrics

3.1 IPX — Interolog Prioritisation Index

The principles underlying our implementation for a prioritisation index are sketched in figure 2 (main document). Both Ensembl and PSICQUIC-enabled PPI databases provide several metadata fields that, taken singularly, can provide useful provenance information and evaluation metrics for specific user needs. As `Bio::Homology::InterologWalk` is meant to be as versatile as possible and should ideally be used to generate a wide range of computational hypotheses, we have thought of fusing several of these indicators together, balancing them so that each piece of evidence contributes to a similar extent. When dealing with the Ensembl-based submodules, we have used their API and the BioPerl API to build supplementary meta-data elements if they were not natively present in the DBs. In the case of Intact, when these indicators were not presented explicitly, we have exploited the meta-data structure (i.e. the PSI-MI ontology) underlying the data to mine supplementary information for our ranking procedure.

Following is a description of the prioritisation features we currently consider. Those related to the two orthology projections are:

•**Orthology Type.** The kind of orthology relationship existing between the two identifiers. This indicates if there is a one-to-one mapping of orthologues, or if in-paralogy events in one or both sides mean we are considering a one-to-many or many-to-many orthologous mapping. We particularly value putative PPIs where *both* orthology relationships are of the one-to-one kind. It has been shown [20] that gene duplication is correlated with sub-functionalisation and neo-functionalisation. When the two orthologous pairs in the interolog walk are of the one-to-one kind we set a boolean variable, Θ, to a non-negative value n in the prioritisation index. We set $\Theta = 0$ for all other pair combinations of orthology types.

•**OPI.** Overall Percentage Identity. A numerical index representing the percentage identity of the *conserved* columns between the two orthology members. It is obtained calling a set of Bioperl methods. The name is actually misleading: this is not an indicator of the overall identity, but rather of the identity between the conserved columns. As shorter sequences are statistically more likely to be conserved than long sequences a full percentage identity indicator would be a poor measure to use. Since we are considering one OPI for each orthology projection in the walk we define a *Joint OPI* as the *geometric mean* of the two, i.e.

$$\mathbf{J}^{(i)}_{\mathrm{OPI}} = \sqrt{\mathrm{OPI}^{(i)}_1 \times \mathrm{OPI}^{(i)}_2} \qquad \forall i. \tag{1}$$

•**Node to Node Distance.** A numerical indicator of the node-to-node distance in the consensus phylogenetic/species tree built by Compara (TreeBeST). We considered node to node distances for both the orthologous pairs in the projection. Assuming that bigger phylogenetic distance corresponds to higher functional divergence, the metric for an interolog is only as good as the biggest of the two distances associated to the orthologous pairs. Normalisation and complement to one yield

$$\mathbf{J}^{(i)}_{\mathrm{nnD}} = 1 - \frac{\max(\mathrm{nnD}^{(i)}_1, \mathrm{nnD}^{(i)}_2)}{\mathrm{nnD}_{\max}} \quad \forall i \tag{2}$$

where $\mathrm{nnD}_{\max}$ represents the highest node-node distance value observed in the dataset of putative PPIs.

•**FSA.** A numerical indicator of the distance between the entry and the orthology pair's First Shared Ancestor in the consensus phylogenetic tree built by Compara/TreeBeST.

•**dN/dS Ratio**. Ratio between the rate of non-synonymous and the rate of synonymous substitutions in the sequences. Also known as the K_a/K_s ratio, it can be interpreted as a measure of the evolutionary pressure acting on two orthologous genes [21, 22]. The following is usually held true:

1. $d_N/d_S \longrightarrow \infty$ strong **positive** selection

Interaction Type	
`MI:0403`	colocalization
`MI:0208`	genetic interaction
`MI:0914`	association
`MI:0915`	physical association
`MI:0407`	direct interaction
**Detection Method**	
`MI:0045`	experimental detection
`MI:0362`	inference
`MI:0063`	interaction prediction
`MI:0686`	unspecified method

Table 1: HUPO PSI-MI 2.5 Ontology Terms used to segregate PPIs in the reference genome.

2. $d_N/d_S \longrightarrow 0$ strong **stabilising** selection
3. $d_N/d_S \approx 1$ some parts of the sequence under positive selection, some under stabilising selections so that overall effects cancel. In some cases, $d_N/d_S \approx 1$ is taken as an indicator for *neutral* selection.

Ensembl only calculates dN/dS values for high-coverage closely-related pairs of species. When the species evolutionary distance is too large, the saturation of the dS values biases the estimated dN/dS ratio. Due to this, while the current release of `Bio::Homology::InterologWalk` collects dN/dS ratios whenever they are present, it ignores their contribution during calculation. Based on the principle that highly conserved protein pairs exist many times also in very diverged organism, we intend to compute and introduce dN/dS ratios as a scoring component in future releases of the software.

As regards the PPI interaction collected from the reference genome, we evaluate the following indicators:

•**Expanded Complex**. Indicates whether the binary interaction has been extracted from a complex using the spoke expansion model. A boolean non-negative term, Σ, is added to the prioritisation index to reward each true binary interaction. $\Sigma = 0$ for spoke-expanded binary interactions.

•**Interaction Type & Interaction Detection Method**. PSI-MI controlled vocabulary terms indicating, respectively, the type of interaction and the detection method used, within HUPO PSI-MI hierarchy. Terms contributing to the prioritisation index are shown in Table 1, pag. 5. If a PPI is annotated with a term that represents a specialisation of those in Table 1, `Bio::Homology::InterologWalk` will climb the hierarchy until one of terms in the table is reached. The PPI will be labelled accordingly.

•**PPI obtained with Multiple Methods & annotated in Multiple Organisms**. (fig. 2D, main document) These two terms acknowledge the fact that an experimental PPI, respectively, reconfirmed through the usage of other detection methods and observed in multiple reference genomes represents sounder evidence. [23–25]

Overall, we define the **Interolog Prioritisation Index** as follows

$$\mathbf{IPX}^{(i)} = \omega_i \left[\mathbf{S}_{\mathrm{PPI}}^{(i)} + \Sigma^{(i)}\right] + \omega_o \left[\mathbf{S}_{\mathrm{ORT}}^{(i)} + \Theta^{(i)}\right] \qquad \forall i \tag{3}$$

where $\mathbf{S}_{\mathrm{PPI}}$ is the contribution to the IPX given by the normalised PPI-related parameters

$$\mathbf{S}_{\mathrm{PPI}}^{(i)} = \frac{i^{(i)}}{\overline{I}_{\mathrm{dir}}} + \frac{d^{(i)}}{\overline{D}_{\mathrm{dir}}} + \frac{m_{\mathrm{dm}}^{(i)}}{\overline{M}_{\mathrm{dir}}} + \frac{m_{\mathrm{taxa}}^{(i)}}{\overline{M}_{\mathrm{taxa}}} \qquad \forall i \tag{4}$$

$\mathbf{S}_{\mathrm{ORT}}$ is the contribution to the IPX given by the orthology-related parameters (eq. 1 and 2)

$$\mathbf{S}_{\mathrm{ORT}}^{(i)} = \mathbf{J}_{\mathrm{OPI}}^{(i)} + \mathbf{J}_{\mathrm{nnD}}^{(i)} \qquad \forall i \tag{5}$$

and ω_i, ω_o, (we set $\omega_i = \omega_o = 1$) are balancing weights for the two contributions. The normalisation parameters in eq. 4 are obtained as follows:

- $\overline{I}_{\text{dir}}$, $\overline{D}_{\text{dir}}$, $\overline{M}_{\text{dir}}$ — mean values computed for the dataset of experimental interaction obtained from the initial gene list;
- $\overline{M}_{\text{taxa}}$ — this parameter cannot be obtained from the dataset of real interactions involving the starting gene set, where no projection to other organisms is involved and no statistics about taxa information is available. In order to normalise $m^{(i)}_{\text{taxa}}$ with a suitable value, `Bio::Homology::InterologWalk` randomly chooses N genomes from the Ensembl pool, and samples m random genes for each of them. For each of the N random gene sets the full interolog walk algorithm is run and putative PPIs are retrieved. A mean $\overline{M}_{\text{taxa}}$ is computed for each so that

$$\overline{M}_{\text{taxa}} = \frac{\overline{M}^{r_1}_{\text{taxa}} + \ldots + \overline{M}^{r_i}_{\text{taxa}} + \ldots \overline{M}^{r_N}_{\text{taxa}}}{N} \tag{6}$$

  where we set $1 \leq m \leq 7$ (using at most 7 well represented taxa to draw random PPIs from) and, if we set G equal to the number of genes in the initial query input file, $N = \min\{500, G\}$.

3.2 PCS — PPI Conservation Score

We also evaluated whether the *structural context* from which each experimental PPI is extracted could provide us with another prioritisation estimate.

Over the past decade, a number of studies have started to draw a connection between protein interaction network topology and protein evolutionary rate. In a seminal paper, Fraser *et al.* [26] used a network of 3541 interactions between 2445 proteins in *S. cerevisiae* to show that the connectivity of well-conserved proteins in the network is negatively correlated with their rate of evolution. As a consequence, more connected proteins evolve at lower rate because they are subject to higher pressure to co-evolve with interacting proteins. In another study, Wuchty *et al.* [27] look at the concept of network *motifs* again in the yeast network and find a correlation between protein evolutionary conservation within a motif and its "interconnectedness".

Based on these results, we decided to implement a score at the purpose of observing the topological context of each experimental PPI used to carry out the interolog walk. The idea is to quantify the level of connectivity of the subnetwork to which each known (direct) PPI interaction participates. Hopefully a binary interaction part of a very well-connected subnetwork in the reference genome is more likely to have retained its functional characterisation after the projection to the organism of interest. One way to do this is to score each reference binary interaction with the *density* value of the most highly-connected subnetwork it participates. This approach draws on a branch of network theory that deals with the concept of γ-*completeness* of a network [28]. A graph $G = (N, E)$, where N is the number of nodes and E is the number of edges, is a γ-*complete graph* $(0 < \gamma \leq 1)$ if every node n_i of G has a degree

$$\deg(n_i) \geq \gamma \cdot (N(G) - 1) \quad \forall i.$$

Given a sub-network with N vertices and E edges we can get its γ-completeness through the density formula,

$$\gamma = \frac{2 \cdot E}{N \cdot (N-1)}. \tag{7}$$

Unfortunately, γ suffers from being biased towards maximally connected small networks. In a biological context it is very unlikely to see complete networks, and it is rather more interesting to recognise and reward networks that are *almost completely* connected ($\gamma \to 1$). In out study, we are interested in spotting binary interactions part of big, quasi-connected sub-networks. In order to account for this, in our implementation we follow the method suggested by [29], where the density term γ is relaxed by weighting it with the number of edges E, to obtain the final **PPI Conservation Score**

$$\textbf{PCS} = \gamma \cdot E = \frac{2 \cdot E^2}{N \cdot (N-1)}. \tag{8}$$

$\mathcal{F}_1$	RP	NRP	*total*
**Mmus-Hsap**	216	56	272
Dmel-Scer	69	220	289
total	285	276	561

$\mathcal{F}_2$	RP	NRP	*total*
**Mmus-Hsap**	216	56	272
Hsap-Dmel	95	436	531
total	311	492	803

$\mathcal{F}_3$	RP	NRP	*total*
**Mmus-Hsap**	216	56	272
Hsap-Scer	89	372	461
total	305	428	733

$\mathcal{F}_4$	RP	NRP	*total*
**Mmus-Hsap**	216	56	272
Scer-Cele	49	96	145
total	265	152	417

Table 2: 2X2 contingency tables for the Fisher Exact Probability Test/Chi-Square Test. Category X (columns): Known Positive Data Retrieval Capability at $\text{IPX}_{\text{thr}} = 15$. Category Y (rows): Known Positive Dataset. RP: Retrieved Known Positive. NRP: Known Positive Not Retrieved.

	**FEPT**		**Chi-Square**	
**Contingency Table**	P (one-tailed)	P (two-tailed)	Yates	Pearsons
$\mathcal{F}_1$	1.69e-41	2.43e-41	170.7	172.91
$\mathcal{F}_2$	3.90e-66	3.90e-66	284.29	286.88
$\mathcal{F}_3$	1.38e-59	1.38e-59	251.9	254.37
$\mathcal{F}_4$	4.16e-20	4.16e-20	83.01	84.97

Table 3: Fisher Exact Probability Test/Chi-Square Test — Results.

4 Validation

4.1 Fisher Test Data for the Positive Retrieval Analysis

Results of the Fisher/Chi-Square tests are presented in table (3). The contingency tables used for the analysis are in table (2). In all 4 instances, the association between rows (biological dataset) and columns (predictive power of the IPX) is considered to be extremely statistically significant.

References

[1] Vilella AJ, Severin J, Ureta-Vidal A, Heng L, Durbin R, Birney E: **EnsemblCompara GeneTrees: Complete, duplication-aware phylogenetic trees in vertebrates**. *Genome Research* 2009, **19**(2):327–335, [http://genome.cshlp.org/content/19/2/327.abstract].

[2] Guindon S, Gascuel O: **A Simple, Fast, and Accurate Algorithm to Estimate Large Phylogenies by Maximum Likelihood**. *Systematic Biology* 2003, **52**(5):696–704, [http://sysbio.oxfordjournals.org/content/52/5/696.abstract].

[3] Sonnhammer ELL, Koonin EV: **Orthology, paralogy and proposed classification for paralog subtypes**. *Trends in Genetics* 2002, **18**(12):619–620, [http://www.sciencedirect.com/science/article/B6TCY-475K105-5/2/ec5032803e4653c42d7165c93fe1c54a].

[4] Rastogi S, Liberles D: **Subfunctionalization of duplicated genes as a transition state to neofunctionalization**. *BMC Evolutionary Biology* 2005, **5**:28, [http://www.biomedcentral.com/1471-2148/5/28].

[5] Koonin EV: **Orthologs, Paralogs, and Evolutionary Genomics**. *Annual Review of Genetics* 2005, **39**:309–338, [http://www.annualreviews.org/doi/abs/10.1146/annurev.genet.39.073003.114725].

[6] Hulsen T, Huynen M, de Vlieg J, Groenen P: **Benchmarking ortholog identification methods using functional genomics data**. *Genome Biology* 2006, **7**(4):R31, [http://genomebiology.com/2006/7/4/R31].

[7] Prieto C, De Las Rivas J: **APID: Agile Protein Interaction DataAnalyzer**. *Nucleic Acids Research* 1 July 2006, **34**(suppl 2):W298–W302, [http://nar.oxfordjournals.org/content/34/suppl_2/W298.abstract].

[8] Razick S, Magklaras G, Donaldson I: **iRefIndex: A consolidated protein interaction database with provenance**. *BMC Bioinformatics* 2008, **9**:405, [http://www.biomedcentral.com/1471-2105/9/405].

[9] Breitkreutz BJ, Stark C, Reguly T, Boucher L, Breitkreutz A, Livstone M, Oughtred R, Lackner DH, Bähler J, Wood V, Dolinski K, Tyers M: **The BioGRID Interaction Database: 2008 update**. *Nucleic Acids Research* 2008, **36**(suppl 1):D637–D640, [http://nar.oxfordjournals.org/content/36/suppl_1/D637.abstract].

[10] Goll J, Rajagopala SV, Shiau SC, Wu H, Lamb BT, Uetz P: **MPIDB: the microbial protein interaction database**. *Bioinformatics* 2008, **24**(15):1743–1744, [http://bioinformatics.oxfordjournals.org/content/24/15/1743.abstract].

[11] Lynn DJ, Winsor GL, Chan C, Richard N, Laird MR, Barsky A, Gardy JL, Roche FM, Chan THW, Shah N, Lo R, Naseer M, Que J, Yau M, Acab M, Tulpan D, Whiteside MD, Chikatamarla A, Mah B, Munzner T, Hokamp K, Hancock REW, Brinkman FSL: **InnateDB: facilitating systems-level analyses of the mammalian innate immune response**. *Mol Syst Biol* 2008, **4**:–, [http://dx.doi.org/10.1038/msb.2008.55].

[12] Chautard E, Ballut L, Thierry-Mieg N, Ricard-Blum S: **MatrixDB, a database focused on extracellular protein–protein and protein–carbohydrate interactions**. *Bioinformatics* 2009, **25**(5):690–691, [http://bioinformatics.oxfordjournals.org/content/25/5/690.abstract].

[13] Jensen LJ, Kuhn M, Stark M, Chaffron S, Creevey C, Muller J, Doerks T, Julien P, Roth A, Simonovic M, Bork P, von Mering C: **STRING 8—a global view on proteins and their functional interactions in 630 organisms**. *Nucleic Acids Research* 2009, **37**(suppl 1):D412–D416, [http://nar.oxfordjournals.org/content/37/suppl_1/D412.abstract].

[14] Matthews L, Gopinath G, Gillespie M, Caudy M, Croft D, de Bono B, Garapati P, Hemish J, Hermjakob H, Jassal B, Kanapin A, Lewis S, Mahajan S, May B, Schmidt E, Vastrik I, Wu G, Birney E, Stein L, D'Eustachio P: **Reactome knowledgebase of human biological pathways and processes**. *Nucleic Acids Research* 2009, **37**(suppl 1):D619–D622, [http://nar.oxfordjournals.org/content/37/suppl_1/D619.abstract].

[15] Aranda B, Achuthan P, Alam-Faruque Y, Armean I, Bridge A, Derow C, Feuermann M, Ghanbarian AT, Kerrien S, Khadake J, Kerssemakers J, Leroy C, Menden M, Michaut M, Montecchi-Palazzi L, Neuhauser SN, Orchard S, Perreau V, Roechert B, van Eijk K, Hermjakob H: **The IntAct molecular interaction database in 2010**. *Nucleic Acids Research* 2010, **38**(suppl 1):D525–D531, [http://nar.oxfordjournals.org/content/38/suppl_1/D525.abstract].

[16] Ceol A, Chatr Aryamontri A, Licata L, Peluso D, Briganti L, Perfetto L, Castagnoli L, Cesareni G: **MINT, the molecular interaction database: 2009 update**. *Nucleic Acids Research* 2010, **38**(suppl 1):D532–D539, [http://nar.oxfordjournals.org/content/38/suppl_1/D532.abstract].

[17] Hermjakob H, Montecchi-Palazzi L, Lewington C, Mudali S, Kerrien S, Orchard S, Vingron M, Roechert B, Roepstorff P, Valencia A, Margalit H, Armstrong J, Bairoch A, Cesareni G, Sherman D, Apweiler R: **IntAct: an open source molecular interaction database**. *Nucleic Acids Research* 2004, **32**(suppl 1):D452–D455, [http://nar.oxfordjournals.org/content/32/suppl_1/D452].

[18] Hermjakob H, Montecchi-Palazzi L, Bader G, Wojcik J, Salwinski L, Ceol A, Moore S, Orchard S, Sarkans U, von Mering C, Roechert B, Poux S, Jung E, Mersch H, Kersey P, Lappe M, Li Y, Zeng R, Rana D, Nikolski M, Husi H, Brun C, Shanker K, Grant SGN, Sander C, Bork P, Zhu W, Pandey A, Brazma A, Jacq B, Vidal M, Sherman D, Legrain P, Cesareni G, Xenarios I, Eisenberg D, Steipe B, Hogue C, Apweiler R: **The HUPO PSI's Molecular Interaction format–a community standard for the representation of protein interaction data**. *Nat Biotech* 2004, **22**(2):177–183, [http://dx.doi.org/10.1038/nbt926].

[19] Kerrien S, Orchard S, Montecchi-Palazzi L, Aranda B, Quinn A, Vinod N, Bader G, Xenarios I, Wojcik J, Sherman D, Tyers M, Salama J, Moore S, Ceol A, Chatr-aryamontri A, Oesterheld M, Stumpflen V, Salwinski L, Nerothin J, Cerami E, Cusick M, Vidal M, Gilson M, Armstrong J, Woollard P, Hogue C, Eisenberg D, Cesareni G, Apweiler R, Hermjakob H: **Broadening the horizon - level 2.5 of the HUPO-PSI format for molecular interactions**. *BMC Biology* 2007, **5**:44, [http://www.biomedcentral.com/1741-7007/5/44].

[20] He X, Zhang J: **Rapid Subfunctionalization Accompanied by Prolonged and Substantial Neofunctionalization in Duplicate Gene Evolution**. *Genetics* 2005, **169**(2):1157–1164, [http://www.genetics.org/cgi/content/abstract/169/2/1157].

[21] Kimura M: **Recent development of the neutral theory viewed from the Wrightian tradition of theoretical population genetics**. *Proceedings of the National Academy of Sciences of the United States of America* 1991, **88**(14):5969–5973, [http://www.pnas.org/content/88/14/5969.abstract].

[22] Yang Z, Bielawski JP: **Statistical methods for detecting molecular adaptation**. *Trends in Ecology & Evolution* 2000, **15**(12):496–503, [http://www.sciencedirect.com/science/article/B6VJ1-41V31XP-F/2/d790d7b9bb9365a9a830eadc5b23f07b].

[23] Matthews LR, Vaglio P, Reboul J, Ge H, Davis BP, Garrels J, Vincent S, Vidal M: **Identification of Potential Interaction Networks Using Sequence-Based Searches for Conserved Protein-Protein Interactions or "Interologs"**. *Genome Research* 2001, **11**(12):2120–2126, [http://genome.cshlp.org/content/11/12/2120.abstract].

[24] von Mering C, Krause R, Snel B, Cornell M, Oliver SG, Fields S, Bork P: **Comparative assessment of large-scale data sets of protein-protein interactions**. *Nature* 2002, **417**(6887):399–403, [http://dx.doi.org/10.1038/nature750].

[25] Lehner B, Fraser A: **A first-draft human protein-interaction map**. *Genome Biology* 2004, **5**(9):R63, [http://genomebiology.com/2004/5/9/R63].

[26] Fraser HB, Hirsh AE, Steinmetz LM, Scharfe C, Feldman MW: **Evolutionary Rate in the Protein Interaction Network**. *Science* 2002, **296**(5568):750–752, [http://www.sciencemag.org/cgi/content/abstract/296/5568/750].

[27] Wuchty S, Oltvai ZN, Barabasi AL: **Evolutionary conservation of motif constituents in the yeast protein interaction network**. *Nat Genet* 2003, **35**(2):176–179, [http://dx.doi.org/10.1038/ng1242].

[28] Bhattacharyya M, Bandyopadhyay S: **Mining the Largest Quasi-clique in Human Protein Interactome**. In *ICAIS '09: Proceedings of the 2009 International Conference on Adaptive and Intelligent Systems*, Washington, DC, USA: IEEE Computer Society 2009:194–199.

[29] Huang TW, Lin CY, Kao CY: **Reconstruction of human protein interolog network using evolutionary conserved network**. *BMC Bioinformatics* 2007, **8**:152, [http://www.biomedcentral.com/1471-2105/8/152].
