# Supplementary material for: Bio::Homology::InterologWalk - A Perl module to build putative protein-protein interaction networks through interolog mapping
Source: BMC Bioinformatics. 2011 Jul 18;12:289. doi: 10.1186/1471-2105-12-289 (PMC3161927; doi:10.1186/1471-2105-12-289)
Supplement: Additional file 6 — Putative sub-network based on 10 core DNA Replication genes. [file 1471-2105-12-289-S6.PDF]

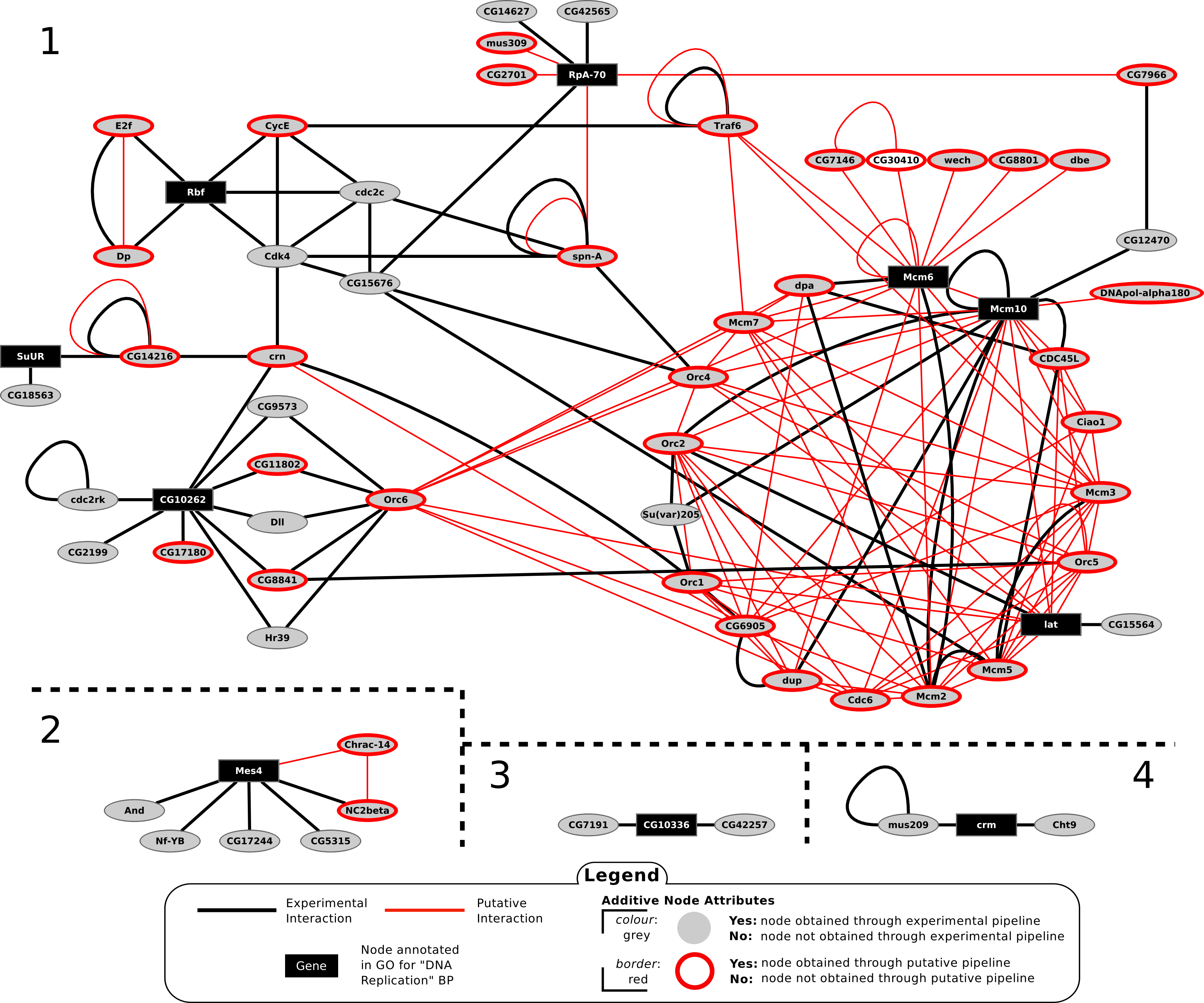
1
CG14627
CG42565
mus309
CG2701
RpA-70
CG7966
E2f
CycE
Traf6
Rbf
cdc2c
CG7146
CG30410
wech
CG8801
dbe
Dp
Cdk4
spn-A
CG15676
CG12470
Mcm6
dpa
Mcm10
DNApol-alpha180
Mcm7
SuUR
CG14216
crn
CDC45L
Orc4
CG18563
CG9573
Ciao1
Orc2
CG11802
Mcm3
cdc2rk
CG10262
Orc6
Su(var)205
Dll
CG2199
CG17180
Orc5
CG8841
Orc1
CG6905
lat
CG15564
Hr39
Mcm5
dup
Cdc6
Mcm2
2
Chrac-14
Mes4
And
NC2beta
Nf-YB
CG17244
CG5315
3
CG7191
CG10336
CG42257
4
mus209
crm
Cht9
Legend
Experimental Interaction
Putative Interaction
Gene
Node annotated in GO for "DNA Replication" BP
Additive Node Attributes
colour: grey
Yes: node obtained through experimental pipeline
No: node not obtained through experimental pipeline
border: red
Yes: node obtained through putative pipeline
No: node not obtained through putative pipeline
